# Artificial Intelligence-Enabled Integration Suggests TP53 Pathway Alterations as Prognostic Biomarkers in Populations with Disproportionate Health Burdens

**DOI:** 10.3390/ijms27031607

**Published:** 2026-02-06

**Authors:** Fernando C. Diaz, Brigette Waldrup, Francisco G. Carranza, Sophia Manjarrez, Enrique Velazquez-Villarreal

**Affiliations:** 1 Lineberger Comprehensive Cancer Center, University of North Carolina, Chapel Hill, NC 27514, USA; 2 Department of Integrative Translational Sciences, Beckman Research Institute, City of Hope, Duarte, CA 91010, USA; 3 City of Hope Comprehensive Cancer Center, Duarte, CA 91010, USA

**Keywords:** early-onset colorectal cancer, TP53 signaling pathway, tumor suppressor, Hispanic/Latino health disparities, FOLFOX chemotherapy, artificial intelligence, AI-agents, biomarkers

## Abstract

The incidence of early-onset colorectal cancer (EOCRC; <50 years) continues to increase, with the most rapid rises occurring among Hispanic/Latino (H/L) populations who remain underrepresented in molecular research. Because the TP53 signaling pathway is a key driver of colorectal tumorigenesis, this study aimed to clarify its prognostic significance in FOLFOX-treated EOCRC across ancestry groups. We analyzed 2515 colorectal cancer (CRC) cases (266 H/L, 2249 non-Hispanic White [NHW]) stratified by ancestry, age at onset, and FOLFOX exposure. Fisher’s exact, chi-square, and Kaplan–Meier’s analyses were applied, and multi-dimensional data integration was performed using AI-HOPE and AI-HOPE-TP53, conversational artificial intelligence platforms enabling natural language-driven exploration of clinical, genomic, and therapeutic features. TP53 pathway alterations were common in both H/L (85%) and NHW (83%) FOLFOX-treated patients. Among late-onset NHW cases, FOLFOX treatment was associated with higher TP53 mutation frequencies and lower ATM and CDKN2A mutation rates compared with untreated counterparts, while CHEK2 alterations were significantly less frequent in late-onset H/L patients. Missense mutations were the predominant alteration type across groups. These findings suggest that TP53 pathway alterations may be associated with ancestry- and treatment-specific clinical patterns in EOCRC and illustrate how AI-enabled integrative analytic frameworks can facilitate hypothesis generation and prioritize candidate biomarkers for future validation in precision oncology.

## 1. Introduction

Colorectal cancer (CRC) ranks as the third leading cause of both cancer-related deaths and incidence in the United States [1]. Although widespread screening and prevention strategies have stabilized or reduced CRC rates in older adults, the disease remains a major public health challenge [2]. Among individuals under 50 years of age—who often fall outside routine screening recommendations—CRC has become the leading cause of cancer-related deaths in men and the second in women [1]. Alarmingly, by 2030, CRC is projected to become the leading cause of cancer-related mortality among individuals aged 20 to 49 [3,4,5,6,7,8,9]. This increase in early-onset colorectal cancer (EOCRC) incidence is most pronounced among Hispanic/Latino (H/L) populations, who have experienced some of the steepest rises in both incidence and mortality in recent years [1,2,6,10,11]. Representing 19% of the U.S. workforce and contributing approximately 15% (~$4.2 trillion) to the U.S. GDP—a figure surpassing the economies of India, the United Kingdom, and France—the H/L population’s growing cancer burden poses an urgent public health and economic challenge [12]. Understanding the molecular drivers of EOCRC in this disproportionately affected group is therefore essential for guiding prevention, therapy optimization, and equitable precision oncology initiatives.

Recent genomic studies have begun to uncover molecular distinctions between EOCRC and late-onset colorectal cancer (LOCRC). Unique features such as LINE-1 hypomethylation and distinct mutational profiles involving SMAD4, *TP53*, *APC*, and *KRAS* suggest that EOCRC may follow a biologically distinct trajectory [13,14,15,16,17]. However, reported differences in tumor mutation burden, microsatellite instability (MSI), and PD-L1 expression remain inconsistent [15,16,17]. Importantly, these investigations have largely focused on homogeneous, non-Hispanic White (NHW) cohorts, leaving significant gaps in the molecular characterization of EOCRC within underrepresented populations [18,19,20]. This lack of diversity in genomic research contributes to persistent health disparities, as findings from one population are often extrapolated to others with differing genetic, environmental, and sociocultural contexts. Our group has recently addressed this gap by defining ancestry-specific alterations in key oncogenic pathways—including WNT, MAPK, JAK/STAT, PI3K, and TP53—in EOCRC among H/L patients [21,22,23]. Yet, the functional and prognostic significance of these molecular features, particularly in the context of treatment response, remains poorly understood.

The *TP53* tumor suppressor, often called the “guardian of the genome,” is the most frequently mutated gene across all cancer types [24]. As the central regulator of the TP53 pathway, it orchestrates cell cycle arrest, apoptosis, DNA repair, and metabolic homeostasis [25,26]. In CRC, *TP53* mutations occur in approximately 74% of tumors, with the majority being missense variants that confer altered or dominant-negative function (27). These mutations have been linked to chemoresistance and poor outcomes in various cancers [24]. Within H/L CRC, our group previously observed higher rates of TP53 alterations compared to NHW patients, although no significant differences were found between early- and late-onset disease within the H/L subgroup [23]. However, the impact of TP53 pathway alterations on treatment outcomes in this population remains unexplored.

For microsatellite-stable (MSS), mismatch repair-proficient (pMMR) metastatic CRC without actionable driver mutations, the ASCO-recommended first-line therapy consists of folinic acid, fluorouracil (5-FU), and oxaliplatin (FOLFOX) [25,27]. While this regimen is a cornerstone of CRC treatment, younger EOCRC patients experience higher toxicity and shorter overall survival compared to their late-onset counterparts [5]. The role of *TP53* in mediating FOLFOX response remains controversial: while some studies suggest improved sensitivity in tumors harboring wild-type *TP53*, others report no consistent correlation between TP53 status and treatment efficacy [28,29,30]. Notably, these analyses have primarily examined European and Asian cohorts, leaving a critical void regarding H/L populations, who may harbor distinct mutational and pharmacogenomic profiles influencing drug response.

In this study, we conducted a comprehensive molecular and survival analysis of the TP53 pathway in early- and late-onset CRC among H/L patients treated with FOLFOX (Figure S1). We further applied AI-HOPE [31] and its specialized module AI-HOPE-TP53 [32], conversational artificial intelligence (AI) systems designed to integrate clinical, genomic, and treatment data through natural language-driven multi-parameter queries. This AI-enabled framework facilitates rapid, reproducible exploration of large-scale datasets, uncovering complex interactions between pathway alterations and clinical outcomes [33,34,35,36,37]. By leveraging this technology, we aim to define the prognostic significance of TP53 pathway alterations and their potential as ancestry- and treatment-specific biomarkers in FOLFOX-treated H/L EOCRC, advancing both precision oncology and health equity in cancer research.

## 2. Results

### 2.1. Baseline Clinical and Demographic Profiles of H/L and NHW CRC Cohorts

Table 1 summarizes the clinical and demographic characteristics of Hispanic/Latino (H/L) and non-Hispanic White (NHW) colorectal cancer (CRC) patients included in this study. Marked disparities in age of onset were evident between groups. Early-onset CRC (EOCRC; <50 years) accounted for 46.9% of all H/L cases but only 30.1% of NHW cases, highlighting a disproportionately younger disease presentation among H/L individuals. Within the EOCRC category, 27.4% of H/L patients received FOLFOX therapy compared with 16.7% of NHW patients, further emphasizing treatment differences across populations. Conversely, late-onset CRC (LOCRC; ≥50 years) was more common among NHW patients (69.9% vs. 53.1% in H/L), and a greater proportion of NHW LOCRC cases received FOLFOX therapy (40.9% vs. 34.2% in H/L).

Cancer type distribution was comparable between groups, with colon adenocarcinoma representing the majority of tumors in both H/L (61.7%) and NHW (59.0%) patients, followed by rectal and colorectal adenocarcinoma subtypes. Sex distribution was also similar, with a modest male predominance observed in both cohorts (59.4% in H/L vs. 56.3% in NHW). Nearly all samples were obtained from primary tumors, ensuring consistent genomic representation across ancestries.

Disease stage at diagnosis revealed comparable distributions, with Stage I–III disease slightly more frequent among H/L patients (58.6% vs. 55.0%), whereas Stage IV disease occurred marginally less often (40.6% vs. 44.7% in NHW). Microsatellite instability (MSI) profiling demonstrated notable ancestry differences: MSI-stable tumors predominated in both groups but were less frequent among H/L patients (75.2% vs. 86.3% in NHW), consistent with previously reported MSI patterns in minority populations. The H/L cohort also had a substantially higher proportion of missing MSI data (13.2% vs. 1.9%), suggesting potential gaps in diagnostic documentation or testing access.

Ethnicity classification further distinguished populations. All NHW patients were annotated as non-Hispanic/non-Spanish, whereas the H/L cohort was primarily composed of individuals categorized as “Spanish/Hispanic/Latino, NOS” (86.5%) or “Mexican/Chicano” (11.3%). These data confirm a well-defined ancestry structure across cohorts.

Collectively, these findings underscore significant ancestry- and age-related differences in CRC presentation, with H/L patients exhibiting higher rates of early-onset disease, lower MSI-stable frequency, and treatment patterns distinct from NHW patients. These patterns reinforce the clinical relevance of ancestry-informed investigations and highlight the need to address disparities in CRC detection, molecular testing, and therapeutic management within underrepresented populations.

### 2.2. Comparative Genomic Analysis by Age and Ancestry

#### 2.2.1. Hispanic/Latino Patients Stratified by Age and FOLFOX Treatment

Table 2A presents clinical and genomic characteristics of H/L CRC patients, analyzed according to age at diagnosis and FOLFOX treatment status. Among early-onset cases, the median age at diagnosis was slightly higher in FOLFOX-treated patients (42 years; IQR 36–47) compared with those who did not receive FOLFOX (40 years; IQR 34–43), with a near-significant trend (*p* = 0.0541). In contrast, among late-onset patients, those treated with FOLFOX were diagnosed at a significantly younger age (59 years; IQR 54–66) relative to non-treated counterparts (62 years; IQR 56–70; *p* = 0.0487). Across both early- and late-onset groups, mutation counts were broadly similar between treatment arms, with no significant differences observed. In EOCRC H/L patients, the tumor mutational burden (TMB) showed a modest, non-significant tendency toward higher values in the non-FOLFOX subgroup. However, in late-onset disease, non-FOLFOX-treated patients displayed a significantly elevated TMB (6.9; IQR 5.6–9.0) compared with FOLFOX-treated individuals (6.1; IQR 4.9–7.8; *p* = 0.0439).

The fraction of genome altered (FGA) was similar across age and treatment groups, indicating that global genomic instability was not affected by FOLFOX exposure. *TP53* mutations were common in both early- and late-onset cohorts (≈78–81%), with no significant association to treatment. Overall, aside from subtle age and TMB differences, mutation burden, genomic alteration patterns, and *TP53* status remained largely consistent across H/L subgroups.

#### 2.2.2. Non-Hispanic White Patients Stratified by Age and FOLFOX Treatment

Table 2B summarizes the clinical and molecular characteristics of NHW CRC patients, stratified by both age group and FOLFOX treatment status. Among early-onset cases, age at diagnosis was comparable between those treated with FOLFOX and those who were not (43 years vs. 44 years; *p* = 0.56), indicating no treatment-related difference in diagnostic timing. However, in the late-onset population, FOLFOX-treated patients were diagnosed at a significantly younger age (63 years; IQR 57–69) compared with their non-treated counterparts (66 years; IQR 57–74; *p* = 4.15 × 10^−7^).

Genomic metrics revealed age- and treatment-dependent patterns. Mutation burden did not differ significantly among early-onset patients, but in late-onset cases, non-FOLFOX patients exhibited a higher median mutation count (8; IQR 6–12) than those receiving FOLFOX (7; IQR 5–9; *p* = 1.22 × 10^−5^). A similar trend was observed for TMB, which remained stable in early-onset disease yet rose significantly among late-onset non-treated patients (6.6 vs. 6.1; *p* = 2.85 × 10^−4^). The FGA was consistent across all groups, showing no association with treatment exposure.

At the gene level, key differences were observed in NHW patients. *TP53* mutations were common across subgroups but occurred less frequently in late-onset FOLFOX-treated cases (68.1% vs. 73.4%; *p* = 0.026). Among DNA damage repair genes, ATM mutations were significantly more frequent in late-onset patients who did not receive FOLFOX (12.1%) compared with those who did (7.2%; *p* = 0.0012). These results suggest that early-onset NHW cases show relatively uniform genomic profiles regardless of therapy, whereas untreated late-onset tumors demonstrate greater mutational complexity.

#### 2.2.3. Ethnic Differences in Early-Onset Colorectal Cancer

Table 2C summarizes EOCRC characteristics in H/L and NHW patients, separated by FOLFOX treatment status. Among patients who received FOLFOX, H/L cases were diagnosed at a slightly younger age than NHW patients (42 vs. 43 years), a difference that did not reach statistical significance (*p* = 0.085). However, in the non-FOLFOX group, the age gap widened considerably, with H/L patients diagnosed significantly earlier (40 vs. 44 years; *p* = 0.0006).

Across both treatment strata, mutation counts and TMB were comparable, although a modest upward trend in TMB was observed among FOLFOX-treated H/L patients (median 6.3 vs. 5.7; *p* = 0.058). The FGA remained consistent between ethnicities, suggesting similar overall genomic instability.

Notable differences emerged in specific gene alterations. TSC1 mutations occurred more frequently in non-FOLFOX-treated H/L patients (11.5%) than in NHW patients (3.3%; *p* = 0.0228), while *RPTOR* mutations showed a comparable enrichment in the same subgroup (7.7% vs. 2.0%; *p* = 0.0443). Conversely, *ATM* mutation rates were similar between treated cohorts but tended to be higher among non-FOLFOX H/L patients (17.3% vs. 7.9%; *p* = 0.059).

These results show that overall mutation load and genomic profiles were similar between early-onset H/L and NHW cases; however, H/L patients presented at younger ages and had higher rates of *TSC1* and *RPTOR* mutations, especially without FOLFOX treatment.

### 2.3. TP53 Pathway Alterations by Age, Ancestry, and Treatment Status

TP53 pathway alteration patterns were analyzed in H/L and NHW CRC cohorts according to patient age at diagnosis and FOLFOX therapy exposure (Table 3A–D).

TP53 pathway alterations were highly prevalent in the H/L cohort and showed no significant association with FOLFOX treatment in either early- or late-onset disease. Among EOCRC patients, alteration rates were 84.9% (treated) and 92.3% (untreated; *p* = 0.27), and among LOCRC patients, 85.7% (treated) and 76.0% (untreated; *p* = 0.22).

Similarly, *TP53* alterations were frequent and treatment-independent in NHW patients, occurring in 82.9% vs. 81.8% of EOCRC cases (treated vs. untreated; *p* = 0.77) and 78.1% vs. 76.4% of LOCRC cases (*p* = 0.46).

Across ancestries, alteration frequencies were comparable. In EOCRC, rates were similar between H/L and NHW patients, both in treated (84.9% vs. 82.9%; *p* = 0.80) and untreated groups (92.3% vs. 81.8%; *p* = 0.06). In LOCRC, alteration prevalence also showed minimal ancestry-related differences, regardless of treatment status. Overall, *TP53* disruption was common across all subgroups and largely unaffected by age, ancestry, or FOLFOX exposure.

### 2.4. Frequencies of Gene Alterations in the TGF-Beta Pathway

#### 2.4.1. Early-Onset Hispanic/Latino Patients

Table S1 summarizes the distribution of TP53 pathway gene mutations in EOCRC H/L patients according to FOLFOX treatment status. TP53 mutations were highly prevalent across both groups—identified in 78.1% of FOLFOX-treated cases and 80.8% of non-treated cases (*p* = 0.89)—indicating no treatment-related difference. Mutations in *CDKN2A* and *CHEK2* were rare, each observed in fewer than 2% of samples, while MDM2 and *MDM4* alterations were absent entirely. A modest enrichment of ATM mutations was noted among patients who did not receive FOLFOX (17.3%) compared with those treated (6.8%), though this trend did not reach statistical significance (*p* = 0.12). Overall, the data suggest that TP53 pathway disruption is widespread, driven primarily by *TP53* itself, with minimal contribution from other regulators such as *MDM2*, *MDM4*, or *CDKN2A*.

#### 2.4.2. Late-Onset Hispanic/Latino Patients

As shown in Table S2, TP53 mutations were the most frequent alterations detected among LOCRC H/L patients, occurring in 78.0% of FOLFOX-treated cases and 72.0% of non-treated cases (*p* = 0.55). Other TP53 pathway components were rarely affected. MDM2 mutations were observed only in the treated group (2.2%), while *MDM4* alterations were completely absent across both subgroups. Similarly, *CDKN2A* and *CHEK2* mutations were infrequent, each identified in ≤1% of cases. *ATM* mutations were slightly more common in untreated patients (12.0%) compared with those receiving FOLFOX (8.8%), though this difference was not statistically significant (*p* = 0.75).

#### 2.4.3. Early-Onset Non-Hispanic White Patients

Table S3 summarizes TP53 pathway mutation frequencies among EOCRC NHW patients stratified by FOLFOX treatment status. TP53 mutations were the most frequent alteration, detected in 78.9% of FOLFOX-treated patients and 75.5% of those who did not receive chemotherapy (*p* = 0.33), showing no significant treatment-related difference. Mutations in *ATM* and *CHEK2* were relatively uncommon, present in 5–8% and 1–3% of cases, respectively, while *CDKN2A* mutations were observed in fewer than 2% of patients. Alterations in *MDM2* and *MDM4* were rare, occurring in less than 2% of cases overall.

#### 2.4.4. Late-Onset Non-Hispanic White Patients

Table S4 summarizes mutation frequencies in TP53 pathway genes among LOCRC NHW patients, stratified by FOLFOX treatment status. *TP53* mutations were the most common event, occurring in 73.4% of FOLFOX-treated and 68.1% of untreated patients—a statistically significant difference (*p* = 0.026) suggesting a modest enrichment among treated cases. Mutations in other pathway regulators were relatively uncommon. ATM alterations were observed more frequently in untreated patients (12.1%) than in those who received FOLFOX (7.2%; *p* = 0.0012), while *CDKN2A* mutations followed a similar pattern (2.5% vs. 1.0%; *p* = 0.036).

#### 2.4.5. Comparison of Early- and Late-Onset Hispanic/Latino Patients Treated with FOLFOX

Table S5 presents the mutational profiles of TP53 pathway genes among H/L CRC patients, stratified by age at diagnosis. *TP53* mutations were detected at nearly identical rates in early-onset (78.1%) and late-onset (78.0%) FOLFOX-treated patients (*p* = 1.00), underscoring the stability of TP53 alteration prevalence across age groups. Mutations in *MDM2*, *CDKN2A*, *ATM*, and *CHEK2* occurred infrequently, each affecting fewer than 10% of cases and showing no significant age-related differences. *MDM4* alterations were entirely absent across both subgroups.

#### 2.4.6. Comparison of Early- and Late-Onset Hispanic/Latino Patients Not Treated with FOLFOX

Table S6 details the distribution of TP53 pathway mutations among untreated H/L CRC patients, stratified by age of onset. *TP53* mutations were highly recurrent in both groups, occurring in 80.8% of early-onset and 72.0% of late-onset patients (*p* = 0.42), indicating no statistically significant age-related difference. Other pathway components showed minimal mutational activity. *ATM* alterations were among the more frequent secondary events, detected in 17.3% of early-onset and 12.0% of late-onset patients, though the difference was not significant (*p* = 0.63).

#### 2.4.7. Comparison of Early- and Late-Onset Non-Hispanic White Patients Treated with FOLFOX

Table S7 summarizes TP53 pathway mutation frequencies in FOLFOX-treated NHW CRC patients, comparing EOCRC and LOCRC groups. TP53 mutations were widespread in both cohorts but appeared slightly more frequent in early-onset patients (78.9%) than in late-onset cases (73.4%), a modest yet statistically significant difference (*p* = 0.046). Alterations in other TP53 regulatory genes were uncommon. *ATM* and *CHEK2* mutations occurred in fewer than 8% and 2% of cases, respectively, while *MDM2*, *MDM4*, and *CDKN2A* alterations were detected in less than 1% of patients overall. A small increase in *MDM2* mutations was observed in early-onset individuals (1.1%) compared with late-onset patients (0.2%), though this did not reach statistical significance (*p* = 0.06).

#### 2.4.8. Early-Onset FOLFOX-Treated Patients: Hispanic/Latino vs. Non-Hispanic White Comparisons

Table S8 shows that TP53 pathway mutations were highly prevalent and nearly identical between FOLFOX-treated EOCRC H/L and NHW patients (78.1% vs. 78.9%; *p* = 1.00). Mutations in *ATM*, *CHEK2*, *CDKN2A*, *MDM2*, and *MDM4* were rare and showed no significant ancestry-related differences. Overall, TP53 pathway profiles were similar across ancestries, with no evidence of divergence in early-onset FOLFOX-treated cases.

#### 2.4.9. Early-Onset Non-FOLFOX Patients: Hispanic/Latino vs. Non-Hispanic White Comparisons

Table S9 compares the prevalence of TP53 pathway mutations between EOCRC H/L and NHW patients who did not receive FOLFOX treatment. *TP53* alterations were the dominant event in both groups, occurring in 80.8% of H/L and 75.5% of NHW patients (*p* = 0.52), indicating similar mutation frequencies across ancestries. Alterations in ATM were more frequent among H/L patients (17.3%) compared with NHW patients (7.9%), a difference approaching borderline significance (*p* = 0.06). Mutations in *CDKN2A* and *CHEK2* were rare (≤2%), while *MDM2* and *MDM4* mutations were absent in H/L patients and occurred in less than 1% of NHW cases.

#### 2.4.10. Late-Onset FOLFOX-Treated Patients: Hispanic/Latino vs. Non-Hispanic White Comparisons

Table S10 presents the distribution of TP53 pathway gene mutations among LOCRC H/L and NHW patients treated with FOLFOX. TP53 mutations were highly prevalent and comparable across ancestries, observed in 73.4% of H/L and 75.5% of NHW patients (*p* = 0.53). Other pathway components showed low mutation frequencies, with no major ancestry-driven differences. Alterations in *ATM*, *MDM2*, *MDM4*, and *CDKN2A* occurred in less than 8% of cases overall and were similarly distributed between the two groups. However, *CHEK2* mutations were slightly enriched in NHW patients (2.6%) compared with H/L patients (0.8%), a statistically significant difference (*p* = 0.022).

#### 2.4.11. Late-Onset Non-FOLFOX Patients: Hispanic/Latino vs. Non-Hispanic White Comparisons

Table S11 compares TP53 pathway alterations between LOCRC H/L and NHW patients who did not receive FOLFOX therapy. TP53 mutations were common in both populations, identified in 72.0% of H/L and 68.1% of NHW patients (*p* = 0.68), indicating comparable mutation burdens across ancestries. Mutations in other pathway genes were infrequent and evenly distributed. ATM alterations, present in roughly 12% of both groups, represented the most recurrent secondary event. *CDKN2A*, *CHEK2*, *MDM2*, and *MDM4* mutations occurred at low frequencies (<3%) and showed no statistically significant ancestry-related differences.

### 2.5. Mutational Landscape of the TP53 Pathway

#### 2.5.1. Early-Onset H/L CRC

**Figure 1:**
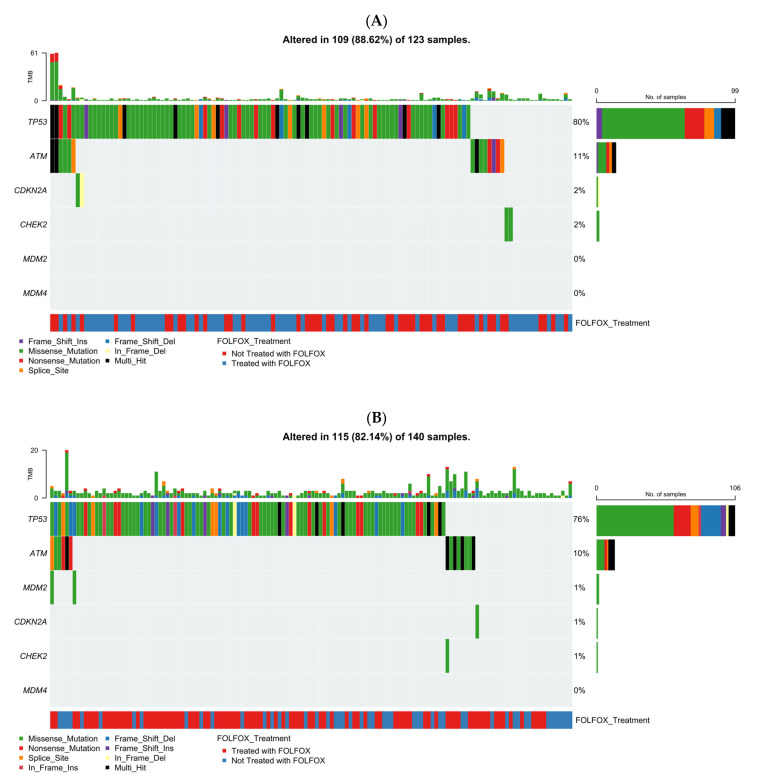

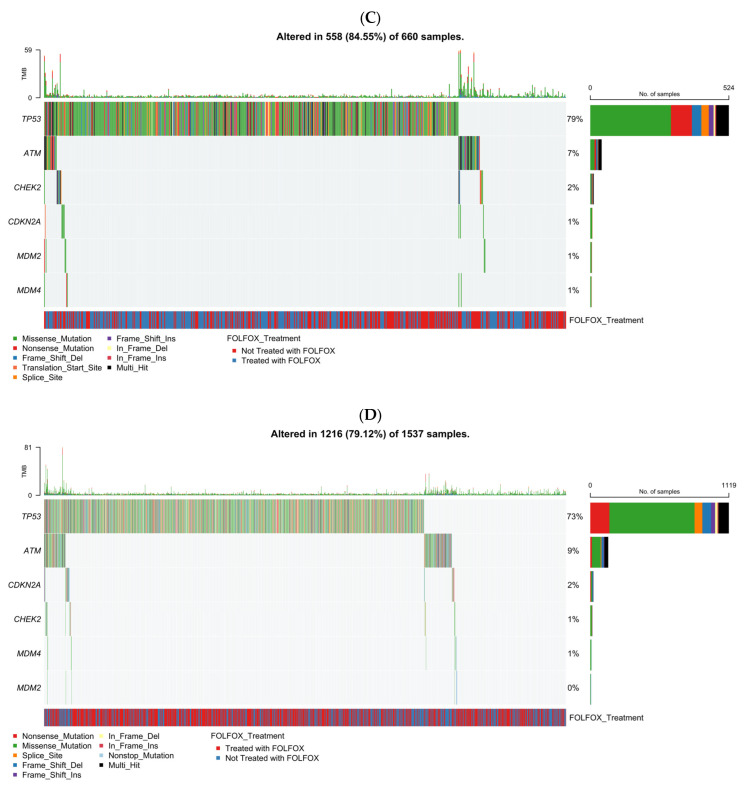
**Somatic mutation landscape of TP53 pathway genes in colorectal cancer (CRC) stratified by age of onset and ancestry.** Oncoplots showing gene-level mutation profiles of key TP53 pathway components in colorectal cancer, stratified by age of onset (early vs. late) and ancestry (Hispanic/Latino vs. Non-Hispanic White). Panels display mutation types, tumor mutational burden (TMB), and FOLFOX treatment status across: (**A**) early-onset Hispanic/Latino (H/L) patients, (**B**) late-onset H/L patients, (**C**) early-onset Non-Hispanic White (NHW) patients, (**D**) late-onset NHW patients.

Figure 1A illustrates the genomic landscape of TP53 pathway alterations in EOCRC H/L patients (n = 123), integrating mutation type, TMB, and FOLFOX treatment status (Table S12). Overall, 88.6% of tumors carried at least one alteration within the TP53 pathway, underscoring its central role in H/L CRC biology. *TP53* emerged as the dominant driver, exhibiting a high frequency of missense mutations (green) accompanied by truncating alterations, including frameshift deletions (light blue) and nonsense mutations (red). Secondary genes such as *ATM* and *CDKN2A* were mutated in smaller subsets (~11% and 2%, respectively), primarily through loss-of-function variants. *CHEK2* alterations were infrequent (~2%), while *MDM2* and *MDM4* remained unaltered across the cohort. TMB levels were generally low, though a few outlier samples displayed elevated mutational burdens without a clear correlation to specific gene alterations. Cases treated with FOLFOX (blue) and untreated (red) were interspersed throughout the oncoplot, suggesting no distinct treatment-associated mutation pattern within the *TP53* signaling network. Collectively, these findings highlight TP53 as the predominant mutational hotspot in early-onset H/L CRC, with co-alterations in DNA repair-related genes contributing modestly to pathway complexity.

#### 2.5.2. Late-Onset H/L CRC

The mutational landscape analysis showed that 82.1% of LOCRC H/L tumors (n = 140) carried TP53 pathway alterations, underscoring persistent pathway disruption in late-onset disease. *TP53* was the most frequently mutated gene, with diverse variant types, followed by less common *ATM* alterations and rare *CDKN2A*, *MDM2*, and *CHEK2* events (<2%); MDM4 mutations were absent. TMB was generally low, and mutation patterns did not differ by FOLFOX status. Overall, TP53 pathway dysregulation was a defining molecular feature in late-onset H/L CRC, with ATM as a recurrent secondary alteration.

#### 2.5.3. Early-Onset NHW CRC

Figure 1C presents the TP53 pathway mutational spectrum in EOCRC NHW patients (n = 660), incorporating mutation type, TMB, and FOLFOX treatment status. Overall, 84.6% of tumors displayed at least one alteration within the *TP53* signaling axis, confirming its pervasive disruption in early-onset disease. *TP53* was the most frequently altered gene, with a predominance of missense mutations (green) interspersed with frameshift deletions (light blue), nonsense mutations (red), and occasional multi-hit events (black). Secondary alterations in *ATM* were relatively common, characterized by truncating and missense variants, whereas mutations in *CDKN2A* and *CHEK2* appeared at much lower frequencies (≤2%). *MDM2* and *MDM4* alterations were exceedingly rare, detected in only isolated cases. TMB values remained largely low across the cohort, although a small subset of hypermutated tumors was observed without clear enrichment for any specific gene. Both FOLFOX-treated (blue) and non-treated (red) patients were evenly distributed along the mutation spectrum, suggesting that chemotherapy exposure did not influence the mutational pattern of *TP53* or its pathway components. Collectively, these data emphasize *TP53* as the principal genomic driver in early-onset NHW CRC, with limited contribution from other pathway regulators.

#### 2.5.4. Late-Onset NHW CRC

The analysis showed that 79.1% of LOCRC NHW tumors (n = 1537) carried TP53 pathway alterations, confirming widespread pathway disruption. TP53 was the most frequently mutated gene, dominated by missense variants, followed by less common alterations in *ATM, CDKN2A*, and *CHEK2; MDM2/MDM4* mutations were rare (<1%). TMB was generally low, with occasional hypermutated outliers. FOLFOX-treated and untreated cases were evenly distributed, indicating no treatment-related mutation differences. Overall, *TP53* was the primary genomic driver in late-onset NHW CRC, with *ATM* and *CDKN2A* contributing modestly.

AI-HOPE [31] and AI-HOPE-TP53 [32] were used to perform exploratory, post-analytic interrogation of the integrated CRC dataset, enabling rapid natural language-driven identification of clinically relevant subgroup differences that informed downstream statistical testing. Among EOCRC patients not treated with FOLFOX, AI-guided queries identified significant ancestry-associated clinical and molecular differences between H/L and NHW groups, including variation in MSI type, cancer type, TP53 status, and age, which were subsequently confirmed by statistical analysis (Figure S3). In LOCRC NHW cases, AI-HOPE-TP53 detected treatment-related differences in TP53 and CDKN2A mutation frequencies between FOLFOX-treated and untreated patients (Figure S4), which aligned with later biostatistical validation. These findings illustrate how AI-based interrogation highlighted subtle but biologically meaningful patterns across treatment and ancestry groups. Overall, AI outputs guided selection of key case–control comparisons, streamlined cohort construction, and supported reproducible integration of clinical, genomic, and treatment variables, forming the analytic foundation for the TP53 pathway mutation landscape presented in this section.

As complementary context to the joint analysis, the TP53 pathway mutational landscape was examined in the largest cohort (MSK-CHORD), illustrating cohort-specific patterns by age of onset and ancestry (Figure S5).

### 2.6. Prognostic Influence of TP53 Pathway Alterations by Age, Ancestry, and Treatment Status

To assess the prognostic significance of TP53 pathway alterations in CRC, we performed Kaplan–Meier’s survival analyses stratified by age at diagnosis, ancestry, and FOLFOX exposure.

**Figure 2:**
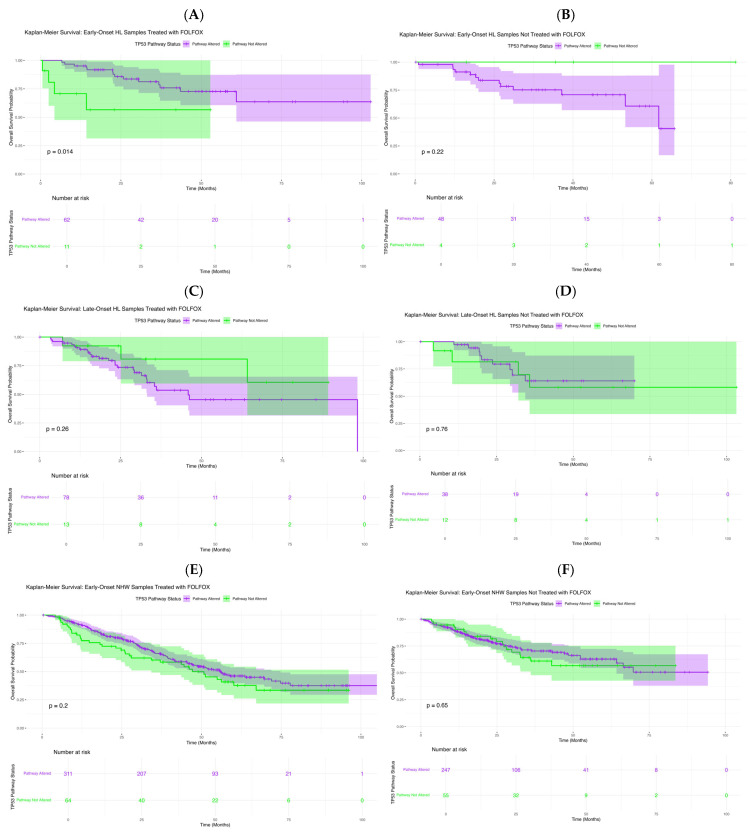
**Kaplan–Meier’s analysis of overall survival by TP53 pathway alteration status across colorectal cancer (CRC) subgroups.** This figure displays survival outcomes stratified by age of onset, ancestry, and FOLFOX treatment. Panels depict: (**A**) Early-Onset Hispanic/Latino (H/L) patients receiving FOLFOX, (**B**) Early-Onset H/L patients not treated with FOLFOX, (**C**) Late-Onset H/L patients treated with FOLFOX, (**D**) Late-Onset H/L patients not treated with FOLFOX, (**E**) Early-Onset Non-Hispanic White (NHW) patients receiving FOLFOX, (**F**) Early-Onset NHW patients not receiving FOLFOX. Each curve compares cases harboring TP53 pathway alterations with those lacking such mutations. Shaded regions represent 95% confidence intervals, and accompanying tables show the number of patients at risk across selected time points.

In exploratory analyses of FOLFOX-treated early-onset Hispanic/Latino patients (Figure 2A), TP53 pathway alterations were associated with differences in overall survival (*p* = 0.014). Those with alterations showed a sharper survival decline within the first 40–50 months, whereas non-altered cases maintained high survival throughout follow-up. Wider confidence intervals among altered cases reflect smaller subgroup size. These findings suggest that TP53 pathway dysregulation may serve as an adverse prognostic factor in early-onset H/L patients receiving FOLFOX. In EOCRC H/L patients not treated with FOLFOX (Figure 2B), survival did not differ significantly by TP53 status (*p* = 0.22). Both subgroups showed favorable, nearly parallel outcomes, and the small late-curve separation lacked statistical significance, likely reflecting limited non-altered cases.

In LOCRC H/L patients treated with FOLFOX (Figure 2C), overall survival did not differ significantly by TP53 pathway status (*p* = 0.26). Although non-altered cases showed slightly higher early survival, curves converged with broad overlap, indicating no clear survival effect. Similarly, in untreated LOCRC H/L patients (Figure 2D), survival was comparable between groups (*p* = 0.76), with overlapping curves and confidence intervals throughout follow-up. These findings suggest that TP53 pathway status has minimal impact on survival in late-onset H/L patients, regardless of FOLFOX exposure.

In EOCRC NHW patients treated with FOLFOX (Figure 2E), overall survival did not differ significantly by TP53 pathway status (*p* = 0.20). Survival curves remained largely parallel, with a brief mid-follow-up divergence that was not statistically meaningful, and overlapping confidence intervals indicated no clear prognostic effect. In untreated EOCRC NHW patients (Figure 2F), survival was similarly unaffected (*p* = 0.65), with closely aligned curves and overlapping intervals throughout follow-up. These results suggest that TP53 pathway alterations do not influence prognosis in early-onset NHW cases, regardless of treatment status.

In LOCRC NHW patients treated with FOLFOX (Figure S6A), TP53 pathway status was not associated with differences in overall survival (*p* = 0.84). Survival curves remained closely aligned with overlapping confidence intervals, indicating no meaningful prognostic effect in this subgroup. Comparably, in untreated LOCRC NHW patients (Figure S6B), survival did not differ significantly by TP53 status (*p* = 0.11). Although a slight separation emerged beyond 60 months, curves and confidence intervals remained largely parallel, suggesting minimal long-term impact of *TP53* alterations in this cohort.

AI-guided subgroup interrogation revealed a potential prognostic distinction linked to TP53 pathway alterations among EOCRC H/L patients treated with FOLFOX. Using the AI-HOPE [31] and AI-HOPE-TP53 [32] frameworks, the analysis compared the case cohort (EO H/L with TP53 pathway alterations, n = 62) against the control cohort (EO H/L without TP53 pathway alterations, n = 11). Subsequent Kaplan–Meier’s survival analysis confirmed that patients harboring TP53 pathway alterations exhibited significantly reduced overall survival compared with non-altered counterparts (log-rank *p* = 0.0139) (Figure S7). The survival trajectories demonstrated an early and sustained separation, with the altered group showing a steeper decline in survival probability over time. These AI-guided results align with the broader survival trends reported in Figure 2B, suggesting that TP53 pathway alterations may represent a negative prognostic biomarker in early-onset H/L CRC patients receiving FOLFOX therapy and underscoring the clinical utility of AI-driven stratification in identifying high-risk molecular subgroups.

## 3. Discussion

In this multi-cohort analysis integrating 2515 CRC across ancestry, age, and treatment strata, we used conventional statistics alongside two conversational AI systems (AI-HOPE and AI-HOPE-TP53) to interrogate how TP53-pathway alterations relate to clinicogenomic features and survival. Two key observations emerge from this analysis. First, disruption of the TP53 pathway is highly prevalent across colorectal cancer, with missense variants predominating in all subgroups, consistent with the well-established central role of *TP53* in CRC biology. Second, treatment-associated shifts in specific pathway components were observed among late-onset non-Hispanic White patients, including enrichment of TP53 alterations and relative depletion of ATM and CDKN2A in FOLFOX-treated cases, suggesting that chemotherapy exposure or treatment selection may influence the detectable genomic landscape. Taken together, these findings suggest that the clinical and prognostic relevance of TP53 pathway alterations may vary depending on therapeutic context, disease characteristics, and population background, warranting further validation.

### 3.1. Context Within the Literature

Prior studies emphasize TP53 as a pervasive driver in CRC and often link *TP53* loss to adverse outcomes or chemoresistance across solid tumors. Yet CRC-specific evidence remains mixed, particularly regarding fluoropyrimidine- and platinum-based regimens. Our ancestry- and treatment-aware stratification adds nuance to this picture. The favorable association of TP53-pathway alterations with overall survival in FOLFOX-treated EO H/L patients contrasts with the commonly assumed negative prognostic role of *TP53*, indicating that (i) the functional consequences of TP53 variants may be context-dependent, (ii) ancestry-linked biology, co-mutation patterns, tumor microenvironmental factors, or pharmacogenomic differences could modulate therapeutic effects of FOLFOX in ways not captured in largely NHW cohorts.

### 3.2. Potential Biological and Clinical Explanations

Several non-mutually exclusive mechanisms may underlie the finding observed in EOCRC H/L patients. One possible explanation involves the spectrum and function of TP53 variants. The relative proportion of missense, loss-of-function, and gain-of-function mutations may differ in EO H/L tumors, and certain missense variants could enhance sensitivity to DNA-damaging agents such as oxaliplatin and 5-fluorouracil, while others promote resistance. Another explanation lies in the co-alteration context. Therapy-associated differences in DNA damage response genes—such as *ATM* and *CDKN2A*—were noted in late-onset NHW cohorts. If EO H/L tumors with TP53-pathway alterations harbor fewer co-mutations in genes that drive chemoresistance or possess distinct cooperating alterations, including the *TSC1* or *RPTOR* variants observed in non-FOLFOX-treated H/L tumors, their overall response to FOLFOX might be enhanced.

Tumor ecology and environmental exposure may also play important roles. Differences in age of onset and ancestry correlate with unique environmental influences, microbiome compositions, and immune profiles, any of which may modify how *TP53*-altered tumors respond to FOLFOX by affecting apoptosis or DNA repair capacity. Additionally, differences in treatment intensity and care delivery may contribute. Younger patients may, on average, tolerate chemotherapy more effectively, and if early-onset Hispanic/Latino patients are more likely to complete or maintain treatment dose intensity, *TP53*-dependent mechanisms could contribute to differential cytotoxic effects of FOLFOX, although this possibility remains speculative and requires further study.

These hypotheses suggest that the interaction between genetic, biological, and treatment-related factors shapes the observed prognostic association in EO H/L CRC. Functional validation using *TP53*-engineered experimental models that recapitulate the variant spectrum found in EO H/L tumors—coupled with oxaliplatin/5-FU response assays and studies incorporating microenvironmental perturbations—will be critical to elucidate the underlying mechanisms.

### 3.3. Implications for Precision Oncology and Health Equity

Our findings argue against a “one-size-fits-all” interpretation of TP53 status in CRC prognosis. Instead, therapy- and ancestry-aware stratification appears essential. For EO H/L patients eligible for FOLFOX, TP53-pathway alteration could help refine prognostication and—pending validation—support treatment counseling. Conversely, the treatment-linked enrichment of TP53 and depletion of *ATM/CDKN2A* in LO NHW cohorts highlight how real-world therapy can bias the genomic snapshots we analyze; clinical sequencing data should be interpreted with treatment history front-of-mind.

More broadly, these results reinforce the need to (i) increase representation of H/L patients in genomic datasets and clinical trials, (ii) integrate ancestry-informed analyses prospectively, (iii) examine pharmacogenomic variants and social determinants of health that could mediate access, adherence, dose intensity, and outcomes. Precision oncology that advances equity must couple molecular insights with context.

### 3.4. Methodological Advances: Conversational AI as an Engine for Discovery

AI-HOPE and AI-HOPE-TP53 accelerated hypothesis generation by enabling natural-language queries that assembled cohorts, surfaced imbalances, and prioritized comparisons for formal testing. This human-in-the-loop approach—rapid exploratory AI followed by orthodox statistics (Fisher’s/χ^2^, KM/log-rank, Cox)—reduced manual data wrangling, improved reproducibility, and exposed subtle patterns (e.g., therapy-linked shifts in *ATM/CDKN2A*) that might otherwise be overlooked. Importantly, AI outputs were treated as pre-statistical leads; only findings that survived conventional inference were highlighted. We view this workflow as a template for scalable, auditable, and equitable biomarker discovery.

### 3.5. Limitations

Several limitations should be considered when interpreting these findings. First, the study’s retrospective and multi-source design introduces inherent variability in treatment documentation and timing. Despite applying stringent criteria to define FOLFOX exposure, residual misclassification cannot be fully excluded. Second, while Cox’s proportional hazards models were adjusted for age, sex, and ancestry, some datasets lacked uniform annotation of important covariates such as disease stage, microsatellite instability status, comorbidities, chemotherapy dose intensity, metastatic sites, and the use of biologic agents. The absence of these variables could introduce unmeasured confounding in the survival analyses. Third, certain early-onset strata—particularly those representing non-altered comparator groups—contained relatively small sample sizes, leading to wider confidence intervals and potential sensitivity to sampling variability.

Additionally, the definition of TP53 pathway alterations was restricted to nonsynonymous mutations in a curated set of genes. Future studies incorporating copy number changes, structural variants, transcriptomic activity scores, and pathway-level functional readouts may provide a more comprehensive understanding of TP53 dysregulation. Finally, while this study integrated multiple publicly available cohorts to increase sample size and diversity, the generalizability of these findings may be limited. Prospective evaluation in larger, more contemporary, and ethnically diverse cohorts, particularly those enriched for EOCRC H/L patients, will be necessary to further assess the potential prognostic and therapeutic relevance of TP53 pathway alterations.

Given the exploratory nature of the analysis, the limited sample size, and the burden of multiple statistical testing, the prognostic association observed in FOLFOX-treated early-onset Hispanic/Latino patients should be interpreted conservatively. As one of the earliest ancestry-resolved studies in early-onset H/L colorectal cancer, the subgroup sizes are necessarily modest, reflecting the broader underrepresentation of minority populations in available genomic datasets. Although the observed survival signal achieved nominal statistical significance, it did not withstand correction for multiple comparisons and should therefore be considered hypothesis-generating rather than conclusive. Nevertheless, the consistency of the directionality, biological plausibility, and treatment-specific context of the findings support a strong rationale for prospective validation. More broadly, these results highlight an ongoing challenge in cancer disparities research: stringent statistical corrections applied to small minority cohorts may mask biologically and clinically meaningful signals. Increasing the representation of Hispanic/Latino patients in national genomic resources and clinical trials will be essential to validate these findings and to advance equitable precision oncology for populations disproportionately impacted by early-onset colorectal cancer.

The observed variation in subgroup size across the dataset necessarily impacts statistical power and interpretability. In Table 2, for example, comparisons involving larger subgroups yielded highly significant *p*-values for age, mutation burden, and TMB; however, the absolute effect sizes were modest (e.g., median 3–4 years in age at diagnosis, a difference of 1 mutation, and 0.5 in TMB). These trends remain informative from an epidemiologic standpoint—particularly age of onset in Hispanic/Latino patients—but may have limited biological or clinical impact when considered in isolation. This highlights an important distinction: statistical significance does not always equate to meaningful effect size, especially in multi-parameter genomic contexts. We therefore interpret these results cautiously and prioritize patterns that persist across analyses, appear clinically plausible, or align with existing literature. Ultimately, larger and more ancestrally diverse datasets will be needed to validate the clinical relevance of these findings and more precisely quantify their effect sizes.

We recognize the value of conducting a cohort-specific meta-analysis with pooled effect estimation to reduce the influence of dataset-level variability and enhance generalizability. However, this approach was not feasible in the present study due to small ancestry-stratified subgroup sizes, particularly among early-onset H/L patients, which limited the statistical power required for independent cohort analyses. To provide additional context without altering the primary analytic framework, we therefore examined the TP53 pathway mutational landscape within the largest cohort included in this study (MSK-CHORD). This cohort-specific analysis was intended to be descriptive, highlighting cohort-specific patterns, and hypothesis-generating. As one of the first ancestry-focused evaluations of TP53 pathway alterations in early-onset colorectal cancer, the available sample sizes reflect broader challenges in genomic data representation for minority populations. We view this limitation as an important call to action: future research should prioritize large-scale, multi-cohort data aggregation efforts intentionally powered for ancestry-specific stratification and appropriate multiple-testing adjustment. Such efforts will be essential to validate these early observations, strengthen confidence in TP53 pathway biomarkers, and more fully characterize treatment- and pathway-linked effects across diverse CRC populations with disproportionate health burdens.

Although TP53 pathway alterations were observed to be associated with overall survival patterns in FOLFOX-treated EOCRC H/L patients in this analysis, these findings should be interpreted conservatively given the study design and analytical constraints. The observed direction of the association differs from the traditionally reported relationship between *TP53* alterations, genomic instability, and adverse prognosis in colorectal cancer. Multiple factors could underlie this observation, including residual confounding, treatment selection effects, and unmeasured clinical variables such as disease stage, treatment intensity, or comorbid conditions, as well as potential ancestry-related biological heterogeneity not captured by bulk genomic profiling. Importantly, the analyses were not intended to establish causal relationships. Accordingly, these results should be viewed as hypothesis-generating and reflective of the complex and context-dependent nature of TP53 pathway alterations in precision oncology. Further investigation using multivariable approaches, prospective study designs, and more granular molecular and treatment-response data, particularly in underrepresented populations, will be necessary to clarify whether TP53 pathway alterations carry ancestry- or therapy-specific prognostic significance in early-onset colorectal cancer.

### 3.6. Future Directions

Future research efforts could consider prospectively evaluating the observed associations between TP53 pathway alterations and clinical outcomes in FOLFOX-treated EOCRC H/L patients. Such studies may benefit from examining *TP53* variant subclasses and co-alteration patterns that could be linked to outcome variability, as well as integrating transcriptional or functional measures of p53 pathway activity, including markers of apoptosis and DNA damage response. Incorporation of pharmacogenomic factors relevant to 5-FU and oxaliplatin metabolism, alongside detailed treatment delivery metrics and social determinants of health, may help to better distinguish biological effects from influences related to access, adherence, or treatment intensity. In the longer term, carefully designed clinical studies could explore stratification by TP53 pathway status within early-onset Hispanic/Latino cohorts to assess whether such molecular features have utility in informing treatment optimization or combination strategies, contingent on supportive evidence from larger and rigorously controlled datasets.

The central objective of this work was to harmonize and analyze clinical–genomic patterns across multiple real-world datasets to capture broader, population-level signals, rather than to compare or contrast cohort-specific effect sizes. Future studies incorporating more evenly balanced cohorts, including underrepresented populations, and prospective designs will be better positioned to formally assess cohort-level heterogeneity using meta-analytic frameworks.

Our findings suggest that the prognostic relevance of TP53 pathway alterations in colorectal cancer may be context dependent. In this analysis, TP53 pathway disruption was associated with overall survival patterns among FOLFOX-treated EOCRC H/L patients, whereas other ancestry-, age-, and treatment-defined groups showed no clear survival association or exhibited treatment-related genomic differences without an evident prognostic signal. By pairing conversational AI with rigorous statistics, we rapidly identified and validated these subgroup effects, illustrating how AI-enabled, equity-minded analytics can uncover clinically actionable patterns that traditional, ancestry-agnostic approaches miss. Prospective validation, multi-omic data integration [37] and mechanistic dissection are now warranted to translate these insights into tailored care for populations most impacted by EOCRC.

## 4. Materials and Methods

### 4.1. Study Design and Data Acquisition

This retrospective multi-cohort analysis integrated publicly available clinical and genomic data from CRC studies hosted on the cBioPortal for Cancer Genomics platform (Table S13). The datasets included TCGA Colorectal Adenocarcinoma (PanCancer Atlas), MSK-CHORD, and AACR Project GENIE BPC CRC (Table S14). These repositories were selected for their detailed annotation of somatic variants, treatment records, and survival outcomes. Only cases with histologically confirmed colorectal, colon, or rectal adenocarcinoma and corresponding primary tumor sequencing data were retained. For individuals represented by multiple tumor entries, a single sample per patient was randomly selected to prevent duplication bias.

### 4.2. Population Stratification and Demographic Classification

Ancestry and ethnicity assignments followed a hierarchical approach prioritizing self-reported categories provided in the original datasets. Patients designated as “Hispanic or Latino,” “Spanish, NOS,” “Hispanic, NOS,” or “Latino, NOS” were classified as H/L. When explicit annotations were missing, surname-based algorithms validated for identifying Hispanic origin were applied. The comparator group included NHW patients meeting identical inclusion criteria. Age at diagnosis was derived from clinical metadata, and participants were grouped as early-onset CRC (EOCRC; <50 years) or late-onset CRC (LOCRC; ≥50 years).

### 4.3. Treatment Annotation and FOLFOX Classification

Therapy data were parsed to identify patients who had received the FOLFOX regimen, consisting of folinic acid (leucovorin), fluorouracil (5-FU), and oxaliplatin. To qualify as “FOLFOX-treated,” treatment timelines were verified to confirm overlapping or sequential administration of all three drugs, consistent with first-line standard-of-care protocols for metastatic, microsatellite-stable CRC. Patients lacking complete documentation of these agents were classified as non-FOLFOX-treated. Treatment records were further cross-checked against clinical notes and regimen codes to ensure internal consistency.

### 4.4. Annotation of TP53 Pathway Alterations

The TP53 signaling network was curated from the peer-reviewed literature and canonical pathway databases, including genes directly involved in cell-cycle regulation, apoptosis, and DNA damage response. Somatic variants were retrieved from cBioPortal mutation calls and filtered to retain only non-synonymous alterations—including missense, nonsense, frameshift, splice-site, and start-loss variants. Pathway alteration status was defined by the presence of at least one qualifying mutation in any TP53 pathway gene. Variants of unknown significance and synonymous mutations were excluded to minimize false-positive associations.

### 4.5. Statistical and Survival Analysis

Differences in mutation frequencies between subgroups were evaluated using Fisher’s exact or chi-square tests, depending on expected cell counts. Continuous clinical variables were analyzed using the Mann–Whitney U test. Overall survival (OS) was calculated from the date of diagnosis to death or last follow-up. Survival distributions were estimated via the Kaplan–Meier method, with log-rank tests used to compare curves. Hazard ratios (HRs) and 95% confidence intervals (CIs) were generated using Cox’s proportional hazards regression, adjusted for age, sex, and ancestry. All analyses were performed in R (v4.3.2), and two-sided *p*-values < 0.05 were considered statistically significant.

Clinical and genomic data were included based on availability at the patient level. As not all cases contained genomic sequencing results, sample sizes differed between clinical summary tables (Table 1) and genomic analyses (e.g., Figure 1B). Inclusion criteria for each dataset type are described explicitly to distinguish clinical-only records from those with molecular data.

To ensure comparability across sources, patient-level variables, including ancestry, age at diagnosis, treatment status, somatic mutation profiles, and survival outcomes, were harmonized using uniform coding and standardized preprocessing pipelines. Because variable availability differed across datasets, sample sizes vary between clinical and genomic analyses. Table 1 summarizes the distribution of key clinical sub-groups stratified by ancestry, age of onset, and FOLFOX exposure. Although cohort-specific heterogeneity in clinical annotation, sequencing methods, and treatment documentation is inherent to multi-source datasets, harmonization enabled consistent integration and subgroup definition. Due to limited ancestry-specific sample size, particularly within early-onset H/L patients, a cohort-level meta-analysis was not feasible; however, ancestry- and treatment-stratified analyses were performed across the combined dataset.

### 4.6. AI-HOPE-TP53 Integration and Automated Analysis Workflow

To streamline high-dimensional data interrogation, we implemented AI-HOPE-TP53, a specialized module within the AI-HOPE (Artificial Intelligence for Health Optimization and Precision Engagement) platform. This conversational AI system enables natural language-driven integration of clinical, genomic, and therapeutic data to execute complex multi-parameter queries and guide downstream analyses. Through iterative prompts such as “Among H/L patients treated with FOLFOX, do TP53 pathway alterations correlate with survival outcomes?” and “Compare mutation frequencies between EOCRC and LOCRC by ancestry and treatment group,” the AI system autonomously generated subgroup stratifications and mutation frequency matrices. Within this analytical framework, AI-HOPE-TP53 performed three interconnected tasks. First, it automatically identified eligible cohorts meeting combined criteria for ancestry, age at onset, treatment exposure, and TP53 pathway alteration status. Second, it generated detailed mutation landscapes, calculating pathway-specific alteration frequencies across defined subgroups. Finally, it conducted outcome association scans to prioritize variables demonstrating significant differences in survival or mutation patterns for formal statistical testing. This AI-assisted workflow minimized manual data handling, enhanced reproducibility, and accelerated the transition from exploratory analyses to validated hypothesis-driven results.

AI-driven analyses were conducted using two conversational artificial intelligence platforms: AI-HOPE [31] and AI-HOPE-TP53 [32]. AI-HOPE [31] served as a global clinical–genomic integrator, enabling natural language-driven cohort construction and exploratory comparisons across patient age, ancestry, and FOLFOX treatment status. This platform facilitated rapid stratification of cases and preliminary identification of clinical and molecular differences between subgroups. In contrast, AI-HOPE-TP53 [32] was designed as a pathway-focused engine used to generate TP53-specific subgroup analyses, mutation frequency matrices, and prognostic associations. Outputs from both platforms were used to guide downstream statistical testing and survival modeling, ensuring that AI-generated insights were validated through conventional biostatistical approaches.

We documented the natural language queries submitted to the AI-HOPE [31] and AI-HOPE-TP53 [32] platforms. These queries guided clinical subgroup construction and hypothesis generation, and the resulting outputs informed downstream statistical and survival analyses. Representative clinical research questions included: (1) “Show me all the meaningful associations between the case and control group.” (Figure S2), which returned AI-derived mutation, clinical, and outcome associations across study subgroups; (2) “Among EO H/L colorectal cancer (CRC) patients treated with FOLFOX, is the TP53 pathway alteration status associated with overall survival (OS)?” (Figure S7), which was associated with differences in overall survival by alteration status; (3) “Among late-onset NHW colorectal cancer (CRC) patients, is there a difference in the frequency of TP53 mutations for those treated and not treated with FOLFOX?” (Figure S3), which produced FOLFOX-dependent mutation patterns; (4) “Among late-onset NHW colorectal cancer (CRC) patients, is there a difference in the frequency of CDKN2A mutations for those treated and not treated with FOLFOX?” (Figure S4), which identified treatment-linked variation in CDKN2A mutations. Each AI-generated result was validated using conventional biostatistical methods and integrated into the final interpretation.

This conversational AI-guided approach reduced manual preprocessing, improved reproducibility, and accelerated translation from exploratory interrogation to validated hypothesis testing, thereby bridging computational precision with biological insight.

## Figures and Tables

**Table 1:** Overview of Hispanic/Latino (H/L) and Non-Hispanic White (NHW) colorectal cancer (CRC) cohorts, including age at diagnosis, FOLFOX treatment status, tumor characteristics, and ancestry.

Clinical Feature	H/L Cohort *n* (%)	NHW Cohort *n* (%)
Age Onset & Treatment		
Early-Onset (<50) Treated with FOLFOX	73 (27.4%)	375 (16.7%)
Late-Onset (≥50) Treated with FOLFOX	91 (34.2%)	919 (40.9%)
Early-Onset (<50) Not Treated with FOLFOX	52 (19.5%)	302 (13.4%)
Late-Onset (≥50) Not Treated with FOLFOX	50 (18.8%)	653 (29.0%)
Cancer Type		
Colon Adenocarcinoma	164 (61.7%)	1328 (59.0%)
Rectal Adenocarcinoma	64 (24.1%)	646 (28.7%)
Colorectal Adenocarcinoma	38 (14.3%)	275 (12.2%)
Sex		
Male	158 (59.4%)	1267 (56.3%)
Female	108 (40.6%)	982 (43.7%)
Sample Type		
Primary Tumor	266 (100.0%)	2249 (100.0%)
Stage at Diagnosis		
Stage 1–3	156 (58.6%)	1236 (55.0%)
Stage 4	108 (40.6%)	1005 (44.7%)
NA	2 (0.8%)	8 (0.4%)
MSI Type		
Stable	200 (75.2%)	1940 (86.3%)
Instable	21 (7.9%)	209 (9.3%)
Indeterminate	10 (3.8%)	57 (2.5%)
NA	35 (13.2%)	43 (1.9%)
Ethnicity		
Spanish NOS; Hispanic NOS, Latino NOS	230 (86.5%)	0 (0.0%)
Mexican (includes Chicano)	30 (11.3%)	0 (0.0%)
Hispanic or Latino	2 (0.8%)	0 (0.0%)
Other Spanish/Hispanic	1 (0.4%)	0 (0.0%)
Spanish surname only	3 (1.1%)	0 (0.0%)
Non-Spanish; Non-Hispanic	0 (0.0%)	2249 (100.0%)

**Table 2:** **Clinical and Genomic Comparison of Early-Onset and Late-Onset Colorectal Cancer (CRC) Cohorts.** It presents an integrated overview of clinical and molecular features distinguishing early-onset (EOCRC) and late-onset (LOCRC) colorectal cancer across major patient subgroups. The analysis encompasses four principal comparisons: (**A**) EOCRC versus LOCRC among Hispanic/Latino (H/L) patients; (**B**) EOCRC versus LOCRC among Non-Hispanic White (NHW) patients; (**C**) cross-ethnic comparison of EOCRC between H/L and NHW cohorts; This comparative framework highlights the interplay between genomic disruption, treatment status, and demographic factors, providing insight into how early disease onset may shape the molecular landscape of CRC across ethnically diverse populations.

(A)						
Clinical Feature	Early-Onset Hispanic/Latino Treated with FOLFOX *n* (%)	Early-Onset Hispanic/Latino Not Treated with FOLFOX *n* (%)	*p*-Value	Late-Onset Hispanic/Latino Treated with FOLFOX *n* (%)	Late-Onset Hispanic/Latino Not Treated with FOLFOX *n* (%)	*p*-Value
Median Diagnosis Age (IQR)	42 (36–47)	40 (34–43)	0.05411	59 (54–66)	62 (56–70)	0.04865
Median Mutation Count	7 (5–8)	7 (5–20)	0.09735	8 (6–9) [NA = 1]	7 (5.25–9)	0.6507
Median TMB (IQR)	6.3 (4.5–7.8) [NA = 15]	5.5 (3.4–8.3) [NA = 2]	0.1719	6.1 (4.9–7.8) [NA = 10]	6.9 (5.6–9.0) [NA = 2]	0.04389
Median FGA	0.18 (0.03–0.27) [NA = 6]	0.19 (0.03–0.29)	0.7661	0.15 (0.06–0.25) [NA = 7]	0.21 (0.04–0.3) [NA = 2]	0.5464
TP53 Mutation						
Present	57 (78.1%)	42 (80.8%)	0.8876	71 (78.0%)	36 (72.0%)	0.5526
Absent	16 (21.9%)	10 (19.2%)		20 (22.0%)	14 (28.0%)	
**(B)**						
**Clinical Feature**	**Early-Onset NHW** **Treated with FOLFOX** ***n* (%)**	**Early-Onset NHW** **Not Treated with FOLFOX** ***n* (%)**	***p*** **-Value**	**Late-Onset NHW** **Treated with FOLFOX** ***n* (%)**	**Late-Onset NHW** **Not Treated with FOLFOX** ***n* (%)**	***p*** **-Value**
Median Diagnosis Age (IQR)	43 (37–48)	44 (38–47)	0.5646	63 (57–69)	66 (57–74)	4.146 × 10^−7^
Median Mutation Count	6 (5–8) [NA = 4]	7 (5–9) [NA = 2]	0.1258	7 (5–9) [NA = 10]	8 (6–12) [NA = 3]	1.22 × 10^−5^
Median TMB (IQR)	5.7 (4.1–6.9)	5.7 (4.1–7.8)	0.4214	6.1 (4.3–8.2)	6.6 (4.9–10.4)	0.0002854
Median FGA	0.14 (0.04–0.24) [NA = 4]	0.15 (0.04–0.23) [NA = 2]	0.5589	0.16 (0.06–0.28) [NA = 6]	0.15 (0.05–0.27) [NA = 5]	0.1929
TP53 Mutation						
Present	296 (78.9%)	228 (75.5%)	0.3319	675 (73.4%)	445 (68.1%)	0.02559
Absent	79 (21.1%)	74 (24.5%)		244 (26.6%)	208 (31.9%)	
CDKN2A Mutation						
Present	3 (0.8%)	5 (1.7%)	0.4772	9 (1.0%)	16 (2.5%)	0.03638
Absent	372 (99.2%)	297 (98.3%)		910 (99.0%)	637 (97.5%)	
ATM Mutation						
Present	19 (5.1%)	24 (7.9%)	0.171	66 (7.2%)	79 (12.1%)	0.001233
Absent	356 (94.9%)	278 (92.1%)		853 (92.8%)	574 (87.9%)	
(C)						
**Clinical Feature**	**Early-Onset Hispanic/Latino** **Treated with FOLFOX** ***n* (%)**	**Early-Onset NHW** **Treated with FOLFOX** ***n* (%)**	***p*** **-Value**	**Early-Onset Hispanic/Latino** **Not Treated with FOLFOX** ***n* (%)**	**Early-Onset NHW** **Not Treated with FOLFOX** ***n* (%)**	***p*** **-Value**
Median Diagnosis Age (IQR)	42 (36–47)	43 (37–48)	0.08467	40 (34–43)	44 (38–47)	0.0006016
Median Mutation Count	7 (5–8)	6 (5–8) [NA = 4]	0.942	7 (5–20)	7 (5–9) [NA = 2]	0.2601
Median TMB (IQR)	6.3 (4.5–7.8) [NA = 15]	5.7 (4.1–6.9)	0.05806	5.5 (3.4–8.3) [NA = 2]	5.7 (4.1–7.8)	0.5732
Median FGA	0.18 (0.03–0.27) [NA = 6]	0.14 (0.04–0.24) [NA = 4]	0.5556	0.19 (0.03–0.29)	0.15 (0.04–0.23) [NA = 2]	0.3612
ATM Mutation						
Present	5 (6.8%)	19 (5.1%)	0.7378	9 (17.3%)	24 (7.9%)	0.05927
Absent	68 (93.2%)	356 (94.9%)		43 (82.7%)	278 (92.1%)	
TSC1 Mutation						
Present	2 (2.7%)	7 (1.9%)	0.6445	6 (11.5%)	10 (3.3%)	0.02282
Absent	71 (97.3%)	368 (98.1%)		46 (88.5%)	292 (96.7%)	
RPTOR Mutation						
Present	2 (2.7%)	10 (2.7%)	1	4 (7.7%)	6 (2.0%)	0.04426
Absent	71 (97.3%)	365 (97.3%)		48 (92.3%)	296 (98.0%)	

**Table 3:** **TP53 Pathway Alterations Across Age, Ancestry, and Treatment Subgroups in Colorectal Cancer.** It presents the distribution of mutations affecting the TP53 signaling pathway among colorectal cancer (CRC) patients, analyzed according to age of onset, ancestry, and FOLFOX treatment status. The analysis is divided into four comparative frameworks: (**A**) early-onset (EOCRC) versus late-onset (LOCRC) within the Hispanic/Latino (H/L) cohort, further separated by treatment exposure; (**B**) FOLFOX-treated versus untreated subgroups within EOCRC and LOCRC among Non-Hispanic White (NHW) patients; (**C**) comparison of EOCRC cases between H/L and NHW populations by treatment status; (**D**) analogous comparison for LOCRC patients. The table details mutation frequencies of major genes implicated in TP53 pathway regulation and evaluates their variation across these clinical contexts. This stratified approach captures potential interactions among age, ancestry, and chemotherapy exposure, revealing how these factors jointly influence TP53 pathway dysregulation and molecular heterogeneity in CRC.

(A)						
Pathway Alterations	Early-Onset Hispanic/Latino Treated with FOLFOX *n* (%)	Early-Onset Hispanic/Latino Not Treated with FOLFOX *n* (%)	*p*-Value	Late-Onset Hispanic/Latino Treated with FOLFOX *n* (%)	Late-Onset Hispanic/Latino Not Treated with FOLFOX *n* (%)	*p*-Value
TP53 Alterations Present	62 (84.9%)	48 (92.3%)	0.2702	78 (85.7%)	38 (76.0%)	0.2246
TP53 Alterations Absent	11 (15.1%)	4 (7.7%)		13 (14.3%)	12 (24.0%)	
**(B)**						
**Pathway Alterations**	**Early-Onset NHW** **Treated with FOLFOX** ***n* (%)**	**Early-Onset NHW** **Not Treated with FOLFOX** ***n* (%)**	***p*** **-Value**	**Late-Onset NHW** **Treated with FOLFOX** ***n* (%)**	**Late-Onset NHW** **Not Treated with FOLFOX** ***n* (%)**	***p*** **-Value**
TP53 Alterations Present	311 (82.9%)	247 (81.8%)	0.7737	718 (78.1%)	499 (76.4%)	0.4601
TP53 Alterations Absent	64 (17.1%)	55 (18.2%)		201 (21.9%)	154 (23.6%)	
**(C)**						
**Pathway Alterations**	**Early-Onset Hispanic/Latino** **Treated with FOLFOX** ***n* (%)**	**Early-Onset NHW** **Treated with FOLFOX** ***n* (%)**	***p*** **-Value**	**Early-Onset Hispanic/Latino** **Not Treated with FOLFOX** ***n* (%)**	**Early-Onset NHW** **Not Treated with FOLFOX** **n (%)**	***p*** **-Value**
TP53 Alterations Present	62 (84.9%)	311 (82.9%)	0.8049	48 (92.3%)	247 (81.8%)	0.06949
TP53 Alterations Absent	11 (15.1%)	64 (17.1%)		4 (7.7%)	55 (18.2%)	
**(D)**						
**Pathway Alterations**	**Late-Onset Hispanic/Latino** **Treated with FOLFOX** ***n* (%)**	**Late-Onset NHW** **Treated with FOLFOX** ***n* (%)**	***p*** **-Value**	**Late-Onset Hispanic/Latino** **Not Treated with FOLFOX** ***n* (%)**	**Late-Onset NHW** **Not Treated with FOLFOX** ***n* (%)**	***p*** **-Value**
TP53 Alterations Present	78 (85.7%)	718 (78.1%)	0.12	38 (76.0%)	499 (76.4%)	1
TP53 Alterations Absent	13 (14.3%)	201 (21.9%)		12 (24.0%)	154 (23.6%)	

## Data Availability

The data presented in this study are openly available in [cBioPortal at https://www.cbioportal.org (accessed on 5 August 2025)] and [genie cBioportal at https://genie.cbioportal.org/ (accessed on 5 August 2025)]. Analytical resources are available through the GitHub repository https://github.com/Velazquez-Villarreal-Lab/AI-TP53 (accessed on 5 August 2025) to promote transparency and reproducibility. Additional data can be provided by the authors upon reasonable request.

## Supplementary Materials

The following supporting information can be downloaded at: https://www.mdpi.com/article/10.3390/ijms27031607/s1.

## References

[1] Siegel R.L., Giaquinto A.N., Jemal A. (2024). Cancer statistics, 2024. CA Cancer J. Clin..

[2] Patel S.G., Karlitz J.J., Yen T., Lieu C.H., Boland C.R. (2022). The rising tide of early-onset colorectal cancer: A comprehensive review of epidemiology, clinical features, biology, risk factors, prevention, and early detection. Lancet Gastroenterol. Hepatol..

[3] Gandini A., Taieb J., Blons H., Netter J., Laurent-Puig P., Gallois C. (2024). Early-Onset colorectal Cancer: From the laboratory to the clinic. Cancer Treat. Rev..

[4] Mauri G., Sartore-Bianchi A., Russo A.G., Marsoni S., Bardelli A., Siena S. (2019). Early-onset colorectal cancer in young individuals. Mol. Oncol..

[5] Meng L., Thapa R., Delgado M.G., Gomez M.F., Ji R., Knepper T.C., Hubbard J.M., Wang X., Permuth J.B., Kim R.D. (2023). Association of Age With Treatment-Related Adverse Events and Survival in Patients with Metastatic Colorectal Cancer. JAMA Netw. Open.

[6] Muller C., Ihionkhan E., Stoffel E.M., Kupfer S.S. (2021). Disparities in Early-Onset Colorectal Cancer. Cells.

[7] Rahib L., Wehner M.R., Matrisian L.M., Nead K.T. (2021). Estimated Projection of US Cancer Incidence and Death to 2040. JAMA Netw. Open.

[8] Santucci C., Mignozzi S., Alicandro G., Pizzato M., Malvezzi M., Negri E., Jha P., La Vecchia C. (2025). Trends in cancer mortality under age 50 in 15 upper-middle and high-income countries. J. Natl. Cancer Inst..

[9] Sinicrope F.A. (2022). Increasing Incidence of Early-Onset Colorectal Cancer. N. Engl. J. Med..

[10] Bhandari A., Woodhouse M., Gupta S. (2017). Colorectal cancer is a leading cause of cancer incidence and mortality among adults younger than 50 years in the USA: A SEER-based analysis with comparison to other young-onset cancers. J. Investig. Med..

[11] Perea J., Martí-Gallostra M., García-Rodríguez A., Vidal-Tocino R., Alcázar J.A., López-Rojo I., Encinas García S., Hurtado E., Jiménez L.M., Álvaro E. (2024). Immigrant Health and Early-Onset Colorectal Cancer Disparities: Results From the Spanish Early-Onset Colorectal Cancer Consortium. JCO Glob Oncol..

[12] Hamilton D.M., Hayes-Bautista D., Hsu P. (2025). 2025 U.S. Latino GDP Report [Internet].

[13] Alshenaifi J.Y., Vetere G., Maddalena G., Yousef M., White M.G., Shen J.P., Vilar E., Parseghian C., Dasari A., Morris V.K. (2025). Mutational and co-mutational landscape of early onset colorectal cancer. Biomarkers.

[14] Antelo M., Balaguer F., Shia J., Shen Y., Hur K., Moreira L., Cuatrecasas M., Bujanda L., Giraldez M.D., Takahashi M. (2012). A high degree of LINE-1 hypomethylation is a unique feature of early-onset colorectal cancer. PLoS ONE.

[15] Lieu C.H., Golemis E.A., Serebriiskii I.G., Newberg J., Hemmerich A., Connelly C., Messersmith W.A., Eng C., Eckhardt S.G., Frampton G. (2019). Comprehensive Genomic Landscapes in Early and Later Onset Colorectal Cancer. Clin. Cancer Res..

[16] Storandt M.H., Shi Q., Eng C., Lieu C., George T., Stoppler M.C., Mauer E., Yilma B., Fragkogianni S., Teslow E.A. (2025). Genomic Landscapes of Early-Onset Versus Average-Onset Colorectal Cancer Populations. Cancers.

[17] Tang J., Peng W., Tian C., Zhang Y., Ji D., Wang L., Jin K., Wang F., Shao Y., Wang X. (2024). Molecular characteristics of early-onset compared with late-onset colorectal cancer: A case controlled study. Int. J. Surg..

[18] Figueroa G.A., Greten T.F., Bonilla C.M. (2024). Unveiling Disparities: Analyzing Hispanic Inclusion in Liver Cancer Research Databases in the United States. J. Racial Ethn. Health Disparities.

[19] Monge C., Greten T.F. (2024). Underrepresentation of Hispanics in clinical trials for liver cancer in the United States over the past 20 years. Cancer Med..

[20] Zavala V.A., Bracci P.M., Carethers J.M., Carvajal-Carmona L., Coggins N.B., Cruz-Correa M.R., Davis M., de Smith A.J., Dutil J., Figueiredo J.C. (2021). Cancer health disparities in racial/ethnic minorities in the United States. Br. J. Cancer.

[21] Monge C., Waldrup B., Carranza F.G., Velazquez-Villarreal E. (2024). WNT and TGF-Beta Pathway Alterations in Early-Onset Colorectal Cancer Among Hispanic/Latino Populations. Cancers.

[22] Monge C., Waldrup B., Carranza F.G., Velazquez-Villarreal E. (2025). Ethnicity-Specific Molecular Alterations in MAPK and JAK/STAT Pathways in Early-Onset Colorectal Cancer. Cancers.

[23] Monge C., Waldrup B., Manjarrez S., Carranza F.G., Velazquez-Villarreal E. (2025). Detecting PI3K and TP53 Pathway Disruptions in Early-Onset Colorectal Cancer Among Hispanic/Latino Patients. Cancer Med..

[24] Sabapathy K., Lane D.P. (2018). Therapeutic targeting of p53: All mutants are equal, but some mutants are more equal than others. Nat. Rev. Clin. Oncol..

[25] O’Reilly M., Linehan A., Krstic A., Kolch W., Sheahan K., Winter D.C., Mc Dermott R. (2023). Oncotherapeutic Strategies in Early Onset Colorectal Cancer. Cancers.

[26] Wang H., Guo M., Wei H., Chen Y. (2023). Targeting p53 pathways: Mechanisms, structures, and advances in therapy. Signal Transduct. Target. Ther..

[27] Morris V.K., Kennedy E.B., Baxter N.N., Benson AB3rd Cercek A., Cho M., Ciombor K.K., Cremolini C., Davis A., Deming D.A., Fakih M.G. (2023). Treatment of Metastatic Colorectal Cancer: ASCO Guideline. J. Clin. Oncol..

[28] Chua W., Goldstein D., Lee C.K., Dhillon H., Michael M., Mitchell P., Clarke S.J., Iacopetta B. (2009). Molecular markers of response and toxicity to FOLFOX chemotherapy in metastatic colorectal cancer. Br. J. Cancer.

[29] Oh H.J., Bae J.M., Wen X., Jung S., Kim Y., Kim K.J., Cho N.Y., Kim J.H., Han S.W., Kim T.Y. (2019). p53 expression status is associated with cancer-specific survival in stage III and high-risk stage II colorectal cancer patients treated with oxaliplatin-based adjuvant chemotherapy. Br. J. Cancer.

[30] Russo A., Bazan V., Iacopetta B., Kerr D., Soussi T., Gebbia N. (2005). TP53-CRC Collaborative Study Group. The TP53 colorectal cancer international collaborative study on the prognostic and predictive significance of p53 mutation: Influence of tumor site, type of mutation, and adjuvant treatment. J. Clin. Oncol..

[31] Yang E.W., Velazquez-Villarreal E. (2025). AI-HOPE: An AI-driven conversational agent for enhanced clinical and genomic data integration in precision medicine research. Bioinformatics.

[32] Yang E.W., Waldrup B., Velazquez-Villarreal E. (2025). AI-HOPE-TP53: A Conversational Artificial Intelligence Agent for Pathway-Centric Analysis of TP53-Driven Molecular Alterations in Early-Onset Colorectal Cancer. Cancers.

[33] Diaz F.C., Waldrup B., Carranza F.G., Manjarrez S., Velazquez-Villarreal E. (2025). Precision Oncology Insights into WNT Pathway Alterations in FOLFOX-Treated Early-Onset Colorectal Cancer in High-Risk Populations. Cancers.

[34] Diaz F.C., Waldrup B., Carranza F.G., Manjarrez S., Velazquez-Villarreal E. (2025). Artificial Intelligence-Enhanced Precision Medicine Reveals Prognostic Impact of TGF-Beta Pathway Alterations in FOLFOX-Treated Early-Onset Colorectal Cancer Among Disproportionately Affected Populations. Int. J. Mol. Sci..

[35] Diaz F.C., Waldrup B., Carranza F.G., Manjarrez S., Velazquez-Villarreal E. (2025). Artificial Intelligence-Guided Molecular Determinants of PI3K Pathway Alterations in Early-Onset Colorectal Cancer Among High-Risk Groups Receiving FOLFOX. Biomedicines.

[36] Yang E.W., Waldrup B., Velazquez-Villarreal E. (2025). Conversational Artificial Intelligence for Integrating Social Determinants, Genomics, and Clinical Data in Precision Medicine: Development and Implementation Study of the AI-HOPE-PM System. JMIR Bioinform. Biotechnol..

[37] Carranza F.G., Diaz F.C., Ninova M., Velazquez-Villarreal E. (2024). Current state and future prospects of spatial biology in colorectal cancer. Front. Oncol..

