# Supplementary material for: Artificial Intelligence-Enabled Integration Suggests TP53 Pathway Alterations as Prognostic Biomarkers in Populations with Disproportionate Health Burdens
Source: Int J Mol Sci. 2026 Feb 6;27(3):1607. doi: 10.3390/ijms27031607 (PMC12898670; doi:10.3390/ijms27031607)
Supplement: Supplementary file 1 [file ijms-27-01607-s001.zip › ijms-4018700-supplementary.pdf]

Supplementary Materials:

Table S1 – Comparison of Early-Onset Hispanic/Latino (H/L) Patients Treated with FOLFOX versus Not Treated with FOLFOX

TP53 Pathway			
**Gene**	**Early-Onset Hispanic/Latino Treated with FOLFOX n (%)**	**Early-Onset Hispanic/Latino Not Treated with FOLFOX n (%)**	**p-value**
**TP53 Mutation**			
Present	57 (78.1%)	42 (80.8%)	0.8876
Absent	16 (21.9%)	10 (19.2%)	
**MDM2 Mutation**			
Present	0 (0.0%)	0 (0.0%)	1
Absent	73 (100.0%)	52 (100.0%)	
**MDM4 Mutation**			
Present	0 (0.0%)	0 (0.0%)	1
Absent	73 (100.0%)	52 (100.0%)	
**CDKN2A Mutation**			
Present	1 (1.4%)	1 (1.9%)	1
Absent	72 (98.6%)	51 (98.1%)	
**ATM Mutation**			
Present	5 (6.8%)	9 (17.3%)	0.1236
Absent	68 (93.2%)	43 (82.7%)	
**CHEK2 Mutation**			
Present	1 (1.4%)	1 (1.9%)	1
Absent	72 (98.6%)	51 (98.1%)	

Table S2 – Comparison of Late-Onset Hispanic/Latino (H/L) Patients Treated with FOLFOX versus Not Treated with FOLFOX

TP53 Pathway			
**Gene**	**Late-Onset Hispanic/Latino Treated with FOLFOX n (%)**	**Late-Onset Hispanic/Latino Not Treated with FOLFOX n (%)**	**p-value**
**TP53 Mutation**			
Present	71 (78.0%)	36 (72.0%)	0.5526
Absent	20 (22.0%)	14 (28.0%)	
**MDM2 Mutation**			
Present	2 (2.2%)	0 (0.0%)	0.539
Absent	89 (97.8%)	50 (100.0%)	
**MDM4 Mutation**			

Present	0 (0.0%)	0 (0.0%)	1
Absent	91 (100.0%)	50 (100.0%)	
**CDKN2A Mutation**			
Present	1 (1.1%)	0 (0.0%)	1
Absent	90 (98.9%)	50 (100.0%)	
**ATM Mutation**			
Present	8 (8.8%)	6 (12.0%)	0.7526
Absent	83 (91.2%)	44 (88.0%)	
**CHEK2 Mutation**			
Present	1 (1.1%)	0 (0.0%)	1
Absent	90 (98.9%)	50 (100.0%)	

Table S3 – Comparison of Early-Onset Non-Hispanic White (NHW) Patients Treated with FOLFOX versus Not Treated with FOLFOX

TP53 Pathway			
**Gene**	**Early-Onset NHW Treated with FOLFOX n (%)**	**Early-Onset NHW Not Treated with FOLFOX n (%)**	**p-value**
**TP53 Mutation**			
Present	296 (78.9%)	228 (75.5%)	0.3319
Absent	79 (21.1%)	74 (24.5%)	
**MDM2 Mutation**			
Present	4 (1.1%)	2 (0.7%)	0.697
Absent	371 (98.9%)	300 (99.3%)	
**MDM4 Mutation**			
Present	1 (0.3%)	4 (1.3%)	0.178
Absent	374 (99.7%)	298 (98.7%)	
**CDKN2A Mutation**			
Present	3 (0.8%)	5 (1.7%)	0.4772
Absent	372 (99.2%)	297 (98.3%)	
**ATM Mutation**			
Present	19 (5.1%)	24 (7.9%)	0.171
Absent	356 (94.9%)	278 (92.1%)	
**CHEK2 Mutation**			
Present	5 (1.3%)	8 (2.6%)	0.3379
Absent	370 (98.7%)	294 (97.4%)	

Table S4 – Comparison of Late-Onset Non-Hispanic White (NHW) Patients Treated with FOLFOX versus Not Treated with FOLFOX

TP53 Pathway			
Gene	Late-Onset NHW Treated with FOLFOX n (%)	Late-Onset NHW Not Treated with FOLFOX n (%)	p-value
**TP53 Mutation**			
Present	675 (73.4%)	445 (68.1%)	**0.02559**
Absent	244 (26.6%)	208 (31.9%)	
**MDM2 Mutation**			
Present	2 (0.2%)	4 (0.6%)	0.2404
Absent	917 (99.8%)	649 (99.4%)	
**MDM4 Mutation**			
Present	4 (0.4%)	5 (0.8%)	0.5023
Absent	915 (99.6%)	648 (99.2%)	
**CDKN2A Mutation**			
Present	9 (1.0%)	16 (2.5%)	**0.03638**
Absent	910 (99.0%)	637 (97.5%)	
**ATM Mutation**			
Present	66 (7.2%)	79 (12.1%)	**0.001233**
Absent	853 (92.8%)	574 (87.9%)	
**CHEK2 Mutation**			
Present	7 (0.8%)	10 (1.5%)	0.2276
Absent	912 (99.2%)	643 (98.5%)	

Table S5 – Comparison of Early-Onset versus Late-Onset Hispanic/Latino (H/L) Patients Treated with FOLFOX

TP53 Pathway			
Gene	Early-Onset Hispanic/Latino Treated with FOLFOX n (%)	Late-Onset Hispanic/Latino Treated with FOLFOX n (%)	p-value
**TP53 Mutation**			
Present	57 (78.1%)	71 (78.0%)	1
Absent	16 (21.9%)	20 (22.0%)	
**MDM2 Mutation**			
Present	0 (0.0%)	2 (2.2%)	0.503
Absent	73 (100.0%)	89 (97.8%)	
**MDM4 Mutation**			
Present	0 (0.0%)	0 (0.0%)	1
Absent	73 (100.0%)	91 (100.0%)	
**CDKN2A Mutation**			
Present	1 (1.4%)	1 (1.1%)	1

Absent	72 (98.6%)	90 (98.9%)	
**ATM Mutation**			
Present	5 (6.8%)	8 (8.8%)	0.8676
Absent	68 (93.2%)	83 (91.2%)	
**CHEK2 Mutation**			
Present	1 (1.4%)	1 (1.1%)	1
Absent	72 (98.6%)	90 (98.9%)	

Table S6 – Comparison of Early-Onset versus Late-Onset Hispanic/Latino (H/L) Patients Not Treated with FOLFOX

**TP53 Pathway**			
**Gene**	**Early-Onset Hispanic/Latino Not Treated with FOLFOX n (%)**	**Late-Onset Hispanic/Latino Not Treated with FOLFOX n (%)**	**p-value**
**TP53 Mutation**			
Present	42 (80.8%)	36 (72.0%)	0.4178
Absent	10 (19.2%)	14 (28.0%)	
**MDM2 Mutation**			
Present	0 (0.0%)	0 (0.0%)	1
Absent	52 (100.0%)	50 (100.0%)	
**MDM4 Mutation**			
Present	0 (0.0%)	0 (0.0%)	1
Absent	52 (100.0%)	50 (100.0%)	
**CDKN2A Mutation**			
Present	1 (1.9%)	0 (0.0%)	1
Absent	51 (98.1%)	50 (100.0%)	
**ATM Mutation**			
Present	9 (17.3%)	6 (12.0%)	0.6334
Absent	43 (82.7%)	44 (88.0%)	
**CHEK2 Mutation**			
Present	1 (1.9%)	0 (0.0%)	1
Absent	51 (98.1%)	50 (100.0%)	

Table S7 – Comparison of Early-Onset versus Late-Onset Non-Hispanic White (NHW) Patients Treated with FOLFOX

**TP53 Pathway**			
**Gene**	**Early-Onset NHW Treated with FOLFOX n (%)**	**Late-Onset NHW Treated with FOLFOX n (%)**	**p-value**

**TP53 Mutation**			
Present	296 (78.9%)	675 (73.4%)	**0.04582**
Absent	79 (21.1%)	244 (26.6%)	
**MDM2 Mutation**			
Present	4 (1.1%)	2 (0.2%)	0.06222
Absent	371 (98.9%)	917 (99.8%)	
**MDM4 Mutation**			
Present	1 (0.3%)	4 (0.4%)	1
Absent	374 (99.7%)	915 (99.6%)	
**CDKN2A Mutation**			
Present	3 (0.8%)	9 (1.0%)	1
Absent	372 (99.2%)	910 (99.0%)	
**ATM Mutation**			
Present	19 (5.1%)	66 (7.2%)	0.2042
Absent	356 (94.9%)	853 (92.8%)	
**CHEK2 Mutation**			
Present	5 (1.3%)	7 (0.8%)	0.5134
Absent	370 (98.7%)	912 (99.2%)	

Table S8 – Comparison of Early-Onset Hispanic/Latino (H/L) versus Early-Onset Non-Hispanic White (NHW) Patients Treated with FOLFOX

**TP53 Pathway**			
**Gene**	**Early-Onset Hispanic/Latino Treated with FOLFOX n (%)**	**Early-Onset NHW Treated with FOLFOX n (%)**	**p-value**
**TP53 Mutation**			
Present	57 (78.1%)	296 (78.9%)	0.995
Absent	16 (21.9%)	79 (21.1%)	
**MDM2 Mutation**			
Present	0 (0.0%)	4 (1.1%)	1
Absent	73 (100.0%)	371 (98.9%)	
**MDM4 Mutation**			
Present	0 (0.0%)	1 (0.3%)	1
Absent	73 (100.0%)	374 (99.7%)	
**CDKN2A Mutation**			
Present	1 (1.4%)	3 (0.8%)	0.5104
Absent	72 (98.6%)	372 (99.2%)	
**ATM Mutation**			
Present	5 (6.8%)	19 (5.1%)	0.7378
Absent	68 (93.2%)	356 (94.9%)	

**CHEK2 Mutation**			
Present	1 (1.4%)	5 (1.3%)	1
Absent	72 (98.6%)	370 (98.7%)	

Table S9 – Comparison of Early-Onset Hispanic/Latino (H/L) versus Early-Onset Non-Hispanic White (NHW) Patients Not Treated with FOLFOX

TP53 Pathway			
**Gene**	**Early-Onset Hispanic/Latino Not Treated with FOLFOX n (%)**	**Early-Onset NHW Not Treated with FOLFOX n (%)**	**p-value**
**TP53 Mutation**			
Present	42 (80.8%)	228 (75.5%)	0.5163
Absent	10 (19.2%)	74 (24.5%)	
**MDM2 Mutation**			
Present	0 (0.0%)	2 (0.7%)	1
Absent	52 (100.0%)	300 (99.3%)	
**MDM4 Mutation**			
Present	0 (0.0%)	4 (1.3%)	1
Absent	52 (100.0%)	298 (98.7%)	
**CDKN2A Mutation**			
Present	1 (1.9%)	5 (1.7%)	1
Absent	51 (98.1%)	297 (98.3%)	
**ATM Mutation**			
Present	9 (17.3%)	24 (7.9%)	0.05927
Absent	43 (82.7%)	278 (92.1%)	
**CHEK2 Mutation**			
Present	1 (1.9%)	8 (2.6%)	1
Absent	51 (98.1%)	294 (97.4%)	

Table S10 – Comparison of Late-Onset Hispanic/Latino (H/L) versus Late-Onset Non-Hispanic White (NHW) Patients Treated with FOLFOX

TP53 Pathway			
**Gene**	**Late-Onset Hispanic/Latino Treated with FOLFOX n (%)**	**Late-Onset NHW Treated with FOLFOX n (%)**	**p-value**
**TP53 Mutation**			
Present	675 (73.4%)	228 (75.5%)	0.5302
Absent	244 (26.6%)	74 (24.5%)	
**MDM2 Mutation**			

Present	2 (0.2%)	2 (0.7%)	0.2571
Absent	917 (99.8%)	300 (99.3%)	
**MDM4 Mutation**			
Present	4 (0.4%)	4 (1.3%)	0.1095
Absent	915 (99.6%)	298 (98.7%)	
**CDKN2A Mutation**			
Present	9 (1.0%)	5 (1.7%)	0.5181
Absent	910 (99.0%)	297 (98.3%)	
**ATM Mutation**			
Present	66 (7.2%)	24 (7.9%)	0.753
Absent	853 (92.8%)	278 (92.1%)	
**CHEK2 Mutation**			
Present	7 (0.8%)	8 (2.6%)	**0.02249**
Absent	912 (99.2%)	294 (97.4%)	

Table S11 – Comparison of Late-Onset Hispanic/Latino (H/L) versus Late-Onset Non-Hispanic White (NHW) Patients Not Treated with FOLFOX

**TP53 Pathway**			
**Gene**	**Late-Onset Hispanic/Latino Not Treated with FOLFOX n (%)**	**Late-Onset NHW Not Treated with FOLFOX n (%)**	**p-value**
**TP53 Mutation**			
Present	36 (72.0%)	445 (68.1%)	0.684
Absent	14 (28.0%)	208 (31.9%)	
**MDM2 Mutation**			
Present	0 (0.0%)	4 (0.6%)	1
Absent	50 (100.0%)	649 (99.4%)	
**MDM4 Mutation**			
Present	0 (0.0%)	5 (0.8%)	1
Absent	50 (100.0%)	648 (99.2%)	
**CDKN2A Mutation**			
Present	0 (0.0%)	16 (2.5%)	0.62
Absent	50 (100.0%)	637 (97.5%)	
**ATM Mutation**			
Present	6 (12.0%)	79 (12.1%)	1
Absent	44 (88.0%)	574 (87.9%)	
**CHEK2 Mutation**			
Present	0 (0.0%)	10 (1.5%)	1
Absent	50 (100.0%)	643 (98.5%)	

Table S12. Distribution of TP53 Pathway Mutation Classes Across Ancestry, Age of Onset, and FOLFOX Treatment in Colorectal Cancer. This table summarizes the proportional distribution of mutation types observed in six key genes of the TP53 signaling pathway, stratified by ancestry [Hispanic/Latino (H/L) vs. Non-Hispanic White (NHW)], disease onset category [early-onset (EO) vs. late-onset (LO)], and FOLFOX chemotherapy status (treated vs. untreated). Mutation classes encompass frame-shift insertions and deletions, in-frame insertions and deletions, missense and nonsense variants, nonstop mutations, splice site and splice region alterations, as well as translation start site changes. The percentages represent the relative contribution of each mutation class within a gene for the corresponding subgroup. Collectively, these data provide insight into the mutational spectrum and allow direct comparison of variant type distributions across ancestry, age, and treatment-defined patient populations.

	Hispanic/Latino Samples				Non-Hispanic White Samples			
	Early-Onset		Late-Onset		Early-Onset		Late-Onset	
	Treated with FOLFOX	Not Treated with FOLFOX	Treated with FOLFOX	Not Treated with FOLFOX	Treated with FOLFOX	Not Treated with FOLFOX	Treated with FOLFOX	Not Treated with FOLFOX
**ATM**								
Frame Shift Deletion	16.7%	0.0%	18.2%	12.5%	10.0%	8.9%	11.0%	12.3%
Frame Shift Insertion	0.0%	5.3%	18.2%	0.0%	3.3%	2.2%	1.2%	11.4%
In Frame Deletion	0.0%	0.0%	0.0%	0.0%	0.0%	0.0%	1.2%	0.0%
Missense Mutation	66.7%	47.4%	54.5%	62.5%	46.7%	75.6%	61.0%	62.3%
Nonsense Mutation	16.7%	36.8%	0.0%	25.0%	36.7%	11.1%	15.9%	12.3%
Splice Site	0.0%	10.5%	9.1%	0.0%	3.3%	2.2%	9.8%	1.8%
**CDKN2A**								
Frame Shift Deletion	0.0%	0.0%	0.0%	0.0%	0.0%	0.0%	20.0%	31.3%
Frame Shift Insertion	0.0%	0.0%	0.0%	0.0%	0.0%	0.0%	10.0%	0.0%

In Frame Deletion	0.0%	100.0%	0.0%	0.0%	0.0%	0.0%	0.0%	0.0%
Missense Mutation	100.0%	0.0%	100.0%	0.0%	100.0%	80.0%	40.0%	43.8%
Nonsense Mutation	0.0%	0.0%	0.0%	0.0%	0.0%	0.0%	30.0%	25.0%
Translation Start Site	0.0%	0.0%	0.0%	0.0%	0.0%	20.0%	0.0%	0.0%
**CHEK2**								
Frame Shift Deletion	0.0%	0.0%	0.0%	0.0%	16.7%	10.0%	0.0%	0.0%
Frame Shift Insertion	0.0%	0.0%	0.0%	0.0%	33.3%	0.0%	28.6%	0.0%
Missense Mutation	100.0%	100.0%	100.0%	0.0%	33.3%	60.0%	71.4%	80.0%
Nonsense Mutation	0.0%	0.0%	0.0%	0.0%	0.0%	20.0%	0.0%	0.0%
Splice Site	0.0%	0.0%	0.0%	0.0%	16.7%	10.0%	0.0%	20.0%
**MDM2**								
Frame Shift Deletion	0.0%	0.0%	0.0%	0.0%	0.0%	0.0%	100.0%	0.0%
Missense Mutation	0.0%	0.0%	100.0%	0.0%	100.0%	50.0%	0.0%	100.0%
Nonsense Mutation	0.0%	0.0%	0.0%	0.0%	0.0%	50.0%	0.0%	0.0%
**MDM4**								

Frame Shift Deletion	0.0%	0.0%	0.0%	0.0%	0.0%	0.0%	0.0%	20.0%
Missense Mutation	0.0%	0.0%	0.0%	0.0%	0.0%	100.0%	100.0%	80.0%
Nonsense Mutation	0.0%	0.0%	0.0%	0.0%	100.0%	0.0%	0.0%	0.0%
**TP53**								
Frame Shift Deletion	6.6%	2.1%	15.3%	10.0%	8.3%	7.6%	6.3%	7.6%
Frame Shift Insertion	3.3%	6.3%	4.2%	2.5%	4.6%	3.6%	3.8%	2.3%
In Frame Deletion	0.0%	0.0%	2.8%	0.0%	1.2%	0.8%	2.3%	1.4%
In Frame Insertion	0.0%	0.0%	2.8%	0.0%	0.6%	0.8%	0.5%	0.8%
Missense Mutation	63.9%	68.8%	54.2%	72.5%	64.4%	64.0%	65.9%	67.8%
Nonsense Mutation	19.7%	14.6%	15.3%	7.5%	16.0%	16.8%	15.3%	13.1%
Nonstop Mutation	0.0%	0.0%	0.0%	0.0%	0.0%	0.0%	0.1%	0.0%
Splice Region	0.0%	0.0%	0.0%	2.5%	0.3%	0.0%	0.1%	0.0%
Splice Site	6.6%	8.3%	5.6%	5.0%	4.6%	6.4%	5.5%	7.0%

Table S13 – TP53 Pathway Genes Included in the Analysis. This table lists the core TP53 pathway genes evaluated in the study and the key references supporting their biological relevance and functional association with TP53 signaling. The gene set includes central regulators (TP53), negative pathway modulators (MDM2, MDM4), cell cycle and tumor suppressor components (CDKN2A), and DNA damage response mediators (ATM, CHEK2). These genes were selected based on established pathway involvement and prior literature evidence (Refs. 23, 30, 32).

Genes evaluated - TP53 pathway	References
TP53	23, 30, 32
MDM2	23, 30, 32
MDM4	23, 30, 32
CDKN2A	23, 30, 32
ATM	23, 30, 32
CHEK2	23, 30, 32

Table S14. Summary characteristics of Hispanic/Latino (H/L) and Non-Hispanic White (NHW) colorectal cancer (CRC) cases across three cohorts (MSK-CHORD, TCGA PanCancer Atlas, and AACR Project GENIE), including age at diagnosis, FOLFOX treatment status, tumor features, and ancestry composition.

	Hispanic/Latino Cohort n (%)			Non-Hispanic White Cohort n (%)	
Clinical Feature	MSK CHORD	TCGA PanCancer Atlas	AACR Project Genie	MSK CHORD	TCGA PanCancer Atlas
**Age Onset & Treatment**					
Early-Onset (< 50) Treated with FOLFOX	57 (24.5%)	1 (50.0%)	15 (48.4%)	374 (16.7%)	1 (12.5%)
Late-Onset (≥ 50) Treated with FOLFOX	80 (34.3%)	1 (50.0%)	10 (32.3%)	916 (40.9%)	3 (37.5%)
Early-Onset (< 50) Not Treated with FOLFOX	48 (20.6%)	0 (0.0%)	4 (12.9%)	301 (13.4%)	1 (12.5%)
Late-Onset (≥ 50) Not Treated with FOLFOX	48 (20.6%)	0 (0.0%)	2 (6.5%)	650 (29.0%)	3 (37.5%)
**Cancer Type**					
Colon Adenocarcinoma	141 (60.5%)	1 (50.0%)	22 (71.0%)	0 (0.0%)	0 (0.0%)
Rectal Adenocarcinoma	56 (24.0%)	1 (50.0%)	7 (22.6%)	0 (0.0%)	0 (0.0%)

Colorectal Adenocarcinoma	36 (15.5%)	2 (100.0%)	0 (0.0%)	2241 (100.0%)	8 (100.0%)
**Sex**					
Male	140 (60.1%)	0 (0.0%)	18 (58.1%)	1263 (56.4%)	4 (50.0%)
Female	93 (39.9%)	2 (100.0%)	13 (41.9%)	978 (43.6%)	4 (50.0%)
**Sample Type**					
Primary Tumor	233 (100.0%)	2 (100.0%)	31 (100.0%)	2241 (100.0%)	8 (100.0%)
**Stage at Diagnosis**					
Stage 1-3	137 (58.8%)	0 (0.0%)	19 (61.3%)	1236 (55.2%)	0 (0.0%)
Stage 4	96 (41.2%)	0 (0.0%)	12 (38.7%)	1005 (44.8%)	0 (0.0%)
NA	0 (0.0%)	2 (100.0%)	0 (0.0%)	0 (0.0%)	8 (100.0%)
**MSI Type**					
Stable	200 (85.8%)	0 (0.0%)	0 (0.0%)	1940 (86.6%)	0 (0.0%)
Instable	21 (9.0%)	0 (0.0%)	0 (0.0%)	209 (9.3%)	0 (0.0%)
Indeterminate	10 (4.3%)	0 (0.0%)	0 (0.0%)	57 (2.5%)	0 (0.0%)
NA	2 (0.9%)	2 (100.0%)	31 (100.0%)	35 (1.6%)	8 (100.0%)
**Ethnicity**					
Spanish NOS; Hispanic NOS, Latino NOS	230 (98.7%)	0 (0.0%)	0 (0.0%)	0 (0.0%)	0 (0.0%)
Mexican (includes Chicano)	2 (0.9%)	0 (0.0%)	28 (90.3%)	0 (0.0%)	0 (0.0%)
Hispanic or Latino	0 (0.0%)	2 (100.0%)	0 (0.0%)	0 (0.0%)	0 (0.0%)
Other Spanish/Hispanic	1 (0.4%)	0 (0.0%)	0 (0.0%)	0 (0.0%)	0 (0.0%)
Spanish surname only	0 (0.0%)	0 (0.0%)	3 (9.7%)	0 (0.0%)	0 (0.0%)
Non-Spanish; Non-Hispanic	0 (0.0%)	0 (0.0%)	0 (0.0%)	2241 (100.0%)	8 (100.0%)

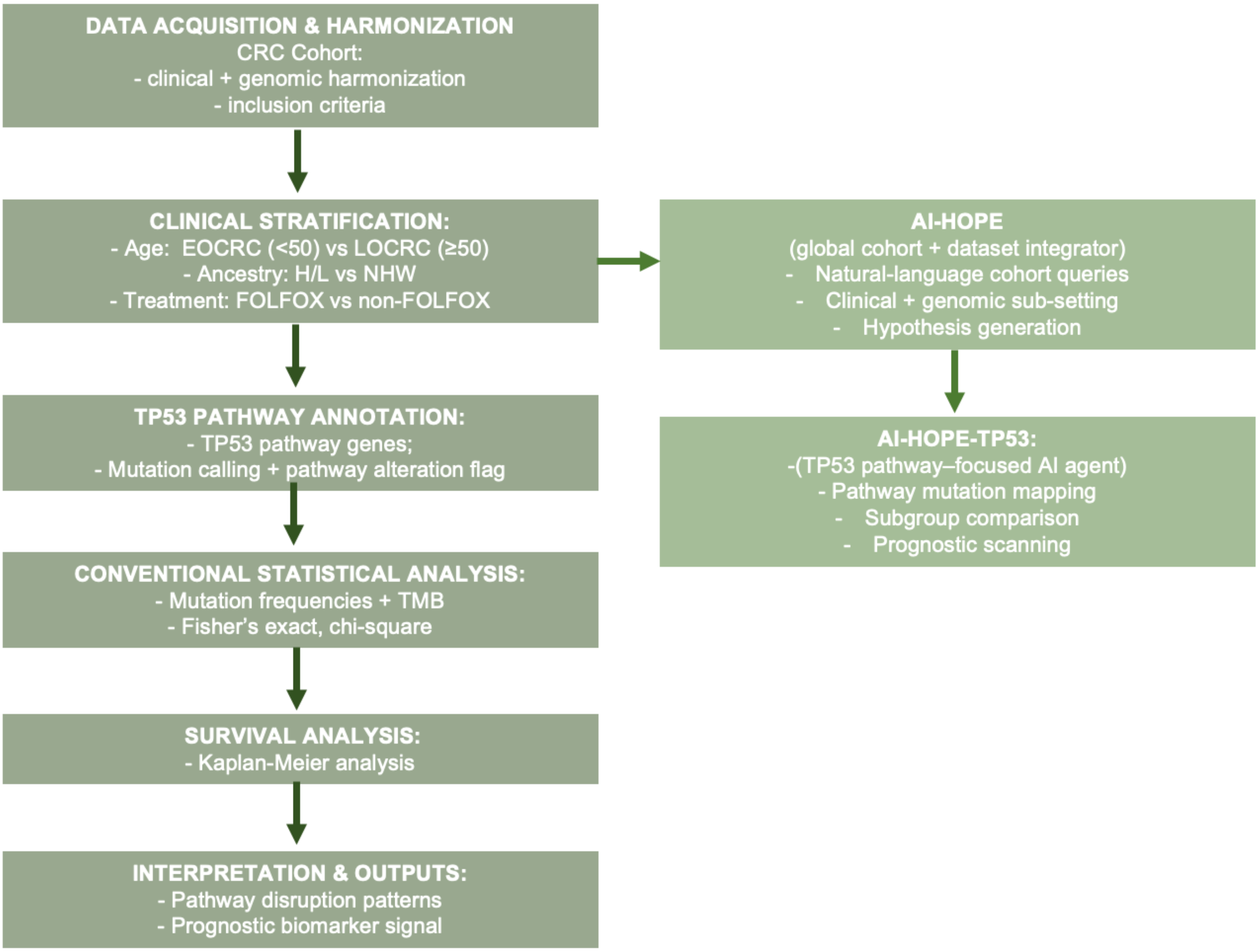

Figure S1. Overall study design and AI-enabled workflow. Public colorectal cancer (CRC) datasets (TCGA, MSK-CHORD, GENIE BPC CRC) were integrated and harmonized, then stratified by ancestry (H/L vs NHW), age of onset (EOCRC vs LOCRC), and FOLFOX treatment status. TP53 pathway alterations were annotated and analyzed using conventional statistics (mutation frequencies, TMB, FGA, and Kaplan–Meier). In parallel, the conversational AI platforms AI-HOPE (global clinical–genomic integrator) and AI-HOPE-TP53 (TP53 pathway–focused agent) were used to perform natural language–driven cohort selection, mutation landscape generation, and outcome association scanning. AI-derived hypotheses and subgroups were then tested with standard biostatistical methods. This integrated pipeline revealed that TP53 pathway alterations act as a favorable prognostic biomarker specifically in FOLFOX-treated early-onset H/L CRC patients, illustrating how AI-guided analytics can accelerate pathway-centric biomarker discovery in precision oncology.

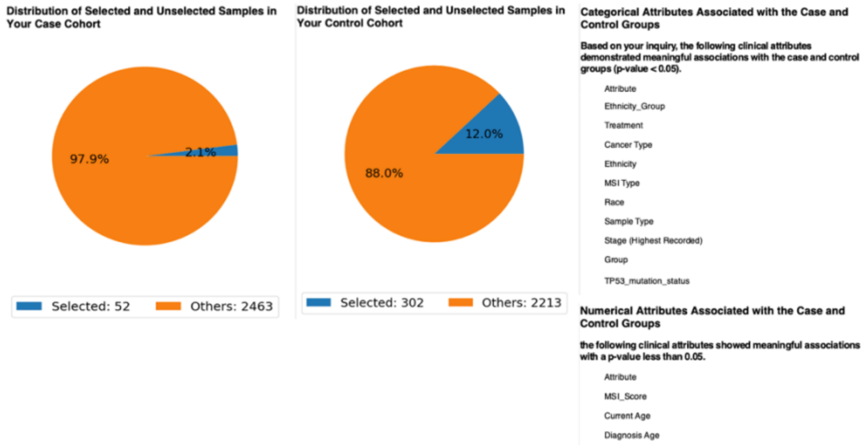

Attribute	Test	Statistic	P-value
Ethnicity	Chi-square		1.35E-77
Ethnicity_Group	Chi-square		3.05E-77
Group	Chi-square		3.05E-77
Race	Chi-square		3.37E-34
TP53_mutation_status	Chi-square		8.34E-22
Stage (Highest Recorded)	Chi-square		2.95E-05
Sample Type	Chi-square		3.52E-05
Cancer Type	Chi-square		0.00015286
MSI_Score	Mann-Whitney U	9468.5	0.00037657
Diagnosis Age	Mann-Whitney U	5513	0.00060157
Current Age	Mann-Whitney U	5402	0.00495165
MSI Type	Chi-square		0.00568395
Treatment	Chi-square		0.00687463

Figure S2. AI-HOPE-TP53-guided identification of significant clinical and molecular attributes distinguishing early-onset Hispanic/Latino (H/L) and Non-Hispanic White (NHW) colorectal cancer (CRC) patients not treated with FOLFOX. The figure summarizes the comparison between the case cohort (early-onset H/L, n = 52) and the control cohort (early-onset NHW, n = 302). Pie charts depict the proportion of selected versus unselected samples in each group. The accompanying tables list all categorical and numerical clinical attributes showing statistically significant differences between the cohorts ($p < 0.05$). Key categorical variables include Ethnicity_Group, Treatment, Cancer Type, Race, MSI Type, TP53 mutation status, Sample Type, and Stage (Highest Recorded). Significant continuous variables include MSI Score, Diagnosis Age, and Current Age. These AI-based analyses reveal multiple layers of divergence between early-onset Hispanic/Latino and Non-Hispanic White CRC patients, emphasizing distinct clinical and molecular profiles relevant to this study.

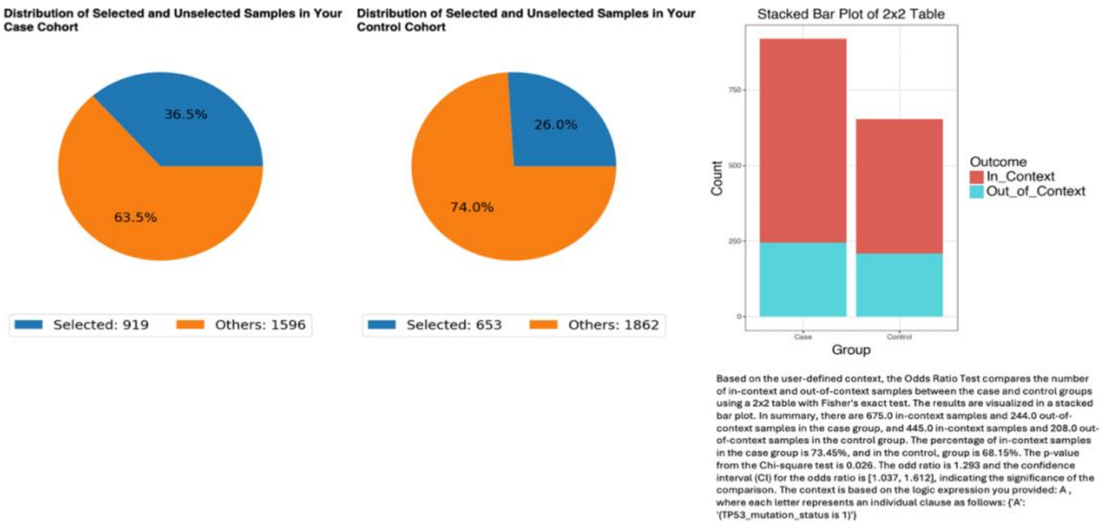

Figure S3. Comparison of TP53 mutation frequency between late-onset Non-Hispanic White (NHW) colorectal cancer (CRC) patients treated versus not treated with FOLFOX. The AI-HOPE and AI-HOPE-TP53 analytical frameworks were used to evaluate the prevalence of TP53 mutations in treatment-stratified late-onset NHW CRC cohorts. (Left) Pie charts illustrate the proportion of selected (in-context) versus unselected (out-of-context) samples in the case cohort—late-onset NHW CRC patients treated with FOLFOX (n = 919)—and the control cohort—late-onset NHW CRC patients not treated with FOLFOX (n = 653). (Right) A stacked bar plot displays the distribution of in-context and out-of-context samples across both groups, analyzed using Fisher's exact test. TP53 mutations were detected in 73.45% of treated and 68.15% of untreated patients, yielding a p-value = 0.026 and an odds ratio = 1.293 (95% CI: 1.037–1.612), indicating a modest but statistically significant enrichment of TP53 mutations in the FOLFOX-treated cohort. These AI-derived findings suggest potential treatment-related selection or biological enrichment of TP53-altered tumors among late-onset NHW CRC patients, supporting the broader mechanistic insights described in this study.

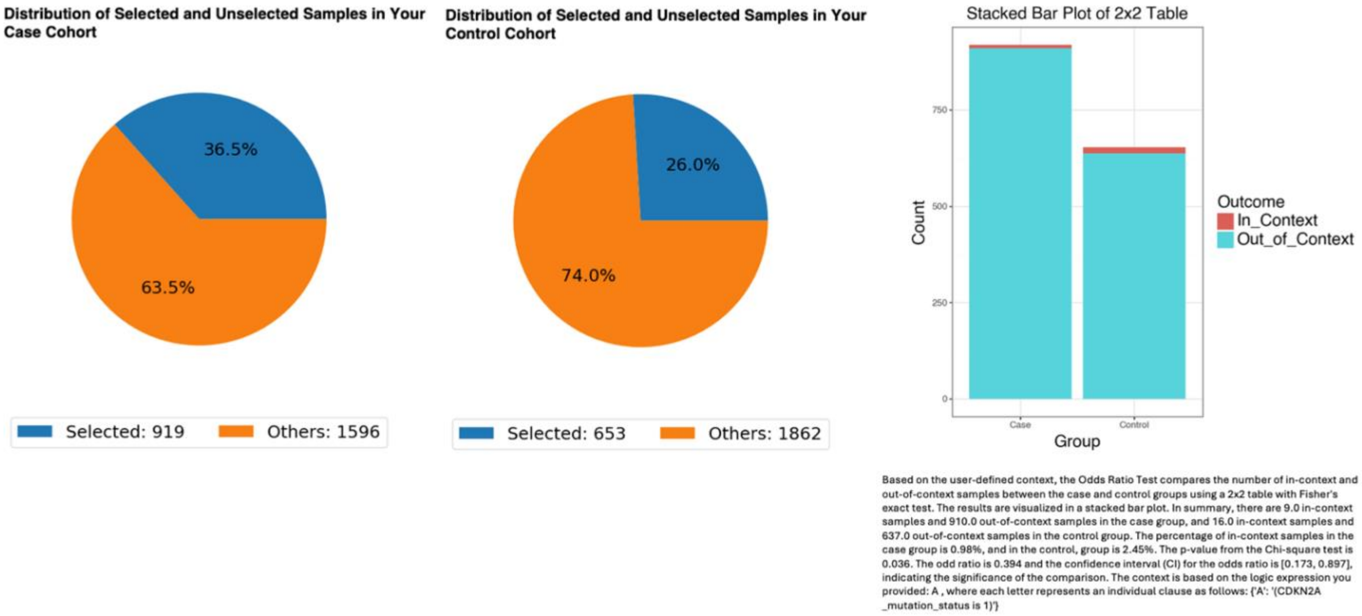

Figure S4. Comparison of CDKN2A mutation frequency between late-onset Non-Hispanic White (NHW) colorectal cancer (CRC) patients treated versus not treated with FOLFOX. The AI-HOPE and AI-HOPE-TP53 analytical frameworks were used to examine the prevalence of CDKN2A mutations among treatment-stratified late-onset NHW CRC cohorts. (Left) Pie charts depict the proportion of selected (in-context) versus unselected (out-of-context) samples in the case cohort—late-onset NHW CRC patients treated with FOLFOX (n = 919)—and the control cohort—late-onset NHW CRC patients not treated with FOLFOX (n = 653). (Right) A stacked bar plot summarizes the number of in-context and out-of-context samples across both groups, analyzed using Fisher's exact test. CDKN2A mutations were observed in 0.98% of treated and 2.45% of untreated patients, yielding a p-value = 0.036 and an odds ratio = 0.394 (95% CI: 0.173–0.897), indicating a significantly lower frequency of CDKN2A mutations among patients treated with FOLFOX. These AI-guided results suggest potential treatment-associated genomic differences in tumor suppressor gene alterations within late-onset NHW CRC, providing additional molecular context for the findings presented in this study.

(A)

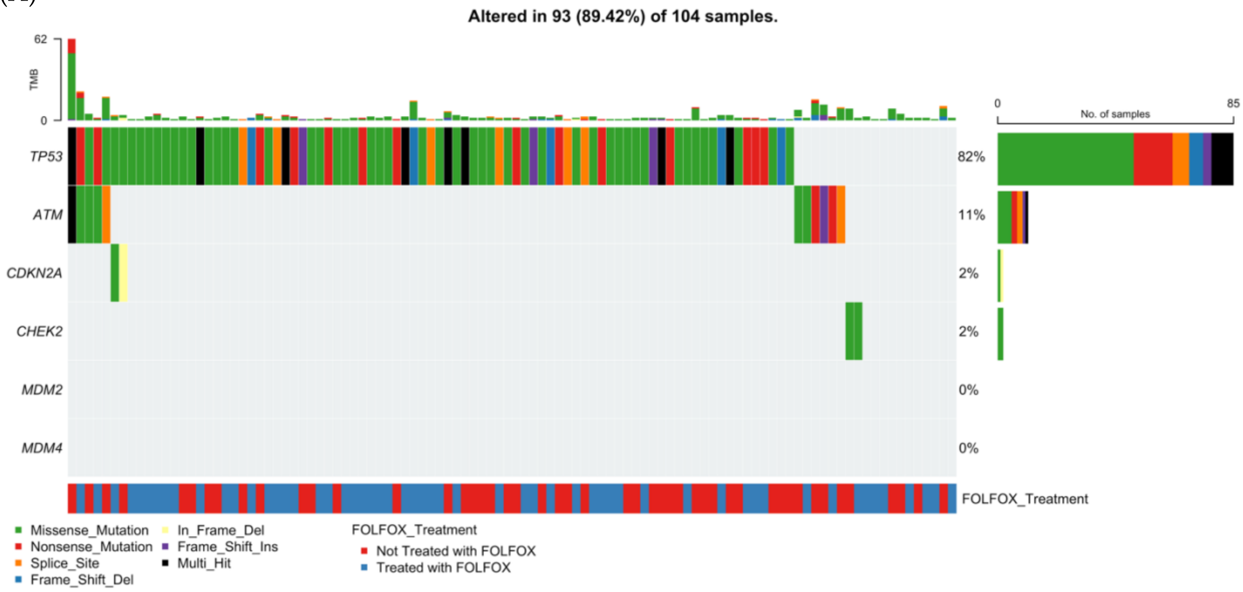
Altered in 93 (89.42%) of 104 samples.
TMB
62
0
TP53
ATM
CDKN2A
CHEK2
MDM2
MDM4
82%
11%
2%
2%
0%
0%
0
No. of samples
85
FOLFOX_Treatment
Missense_Mutation
Nonsense_Mutation
Splice_Site
Frame_Shift_Del
In_Frame_Del
Frame_Shift_Ins
Multi_Hit
FOLFOX_Treatment
Not Treated with FOLFOX
Treated with FOLFOX

(B)

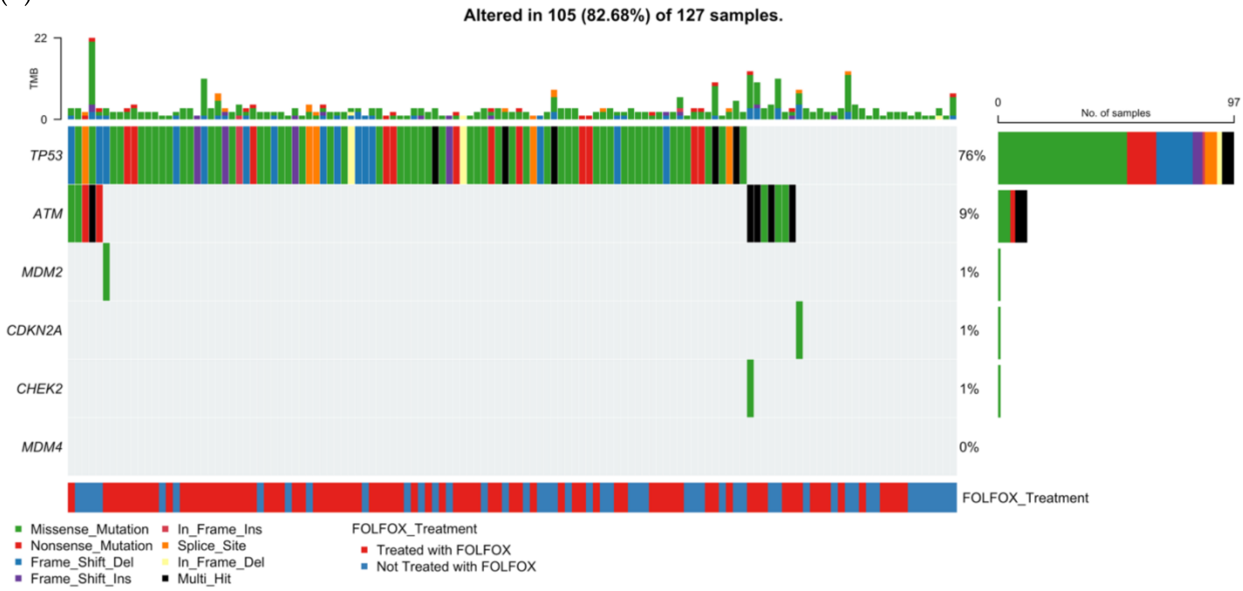
Altered in 105 (82.68%) of 127 samples.
TMB
22
0
TP53
ATM
MDM2
CDKN2A
CHEK2
MDM4
76%
9%
1%
1%
1%
0%
0
No. of samples
97
FOLFOX_Treatment
Missense_Mutation
Nonsense_Mutation
Frame_Shift_Del
Frame_Shift_Ins
In_Frame_Ins
Splice_Site
In_Frame_Del
Multi_Hit
FOLFOX_Treatment
Treated with FOLFOX
Not Treated with FOLFOX

(C)

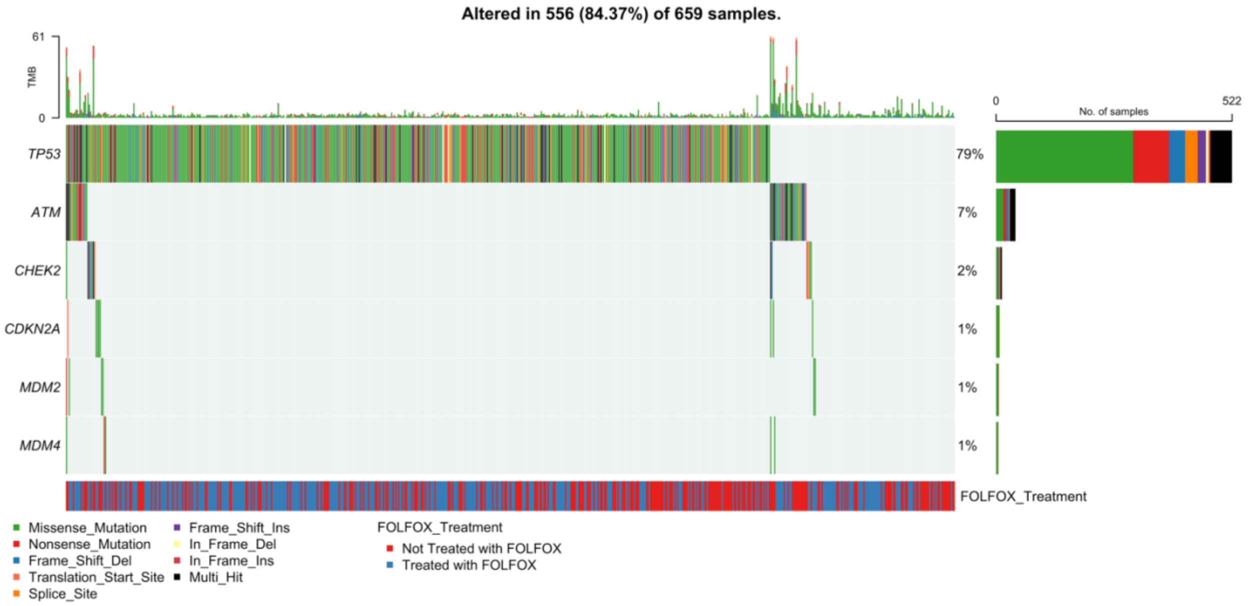

(D)

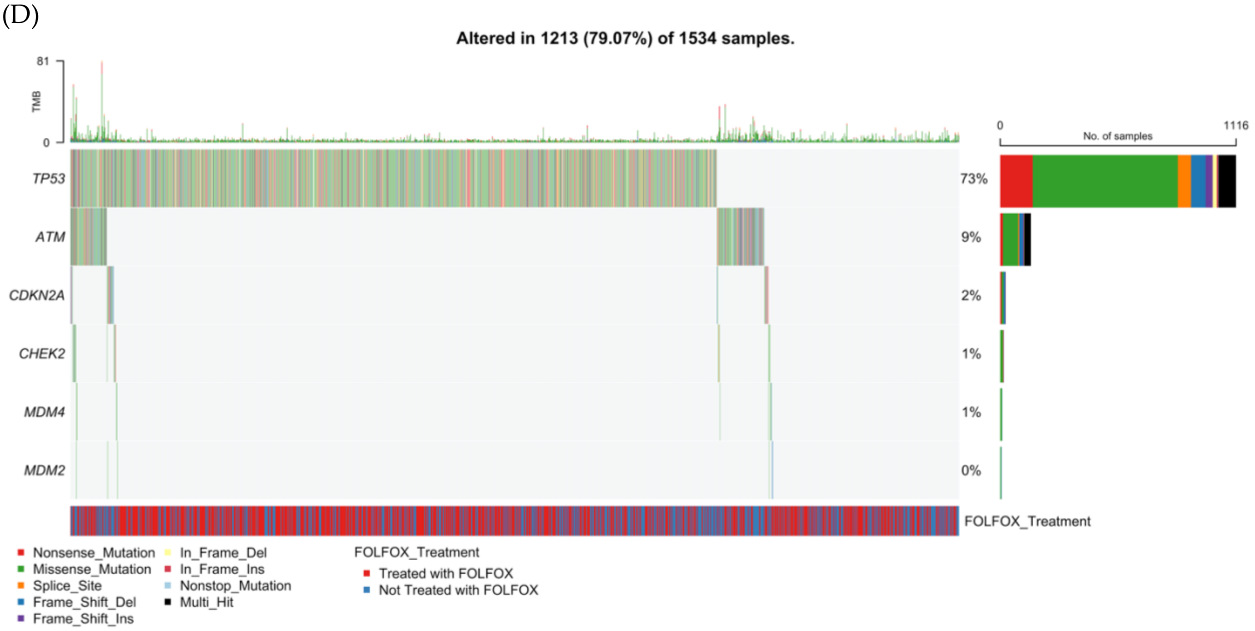

Figure S5. Somatic mutation landscape of TP53 pathway genes in colorectal cancer (CRC) stratified by age of onset and ancestry using the largest cohort (MSK CHORD) in the study. Oncoplots showing gene-level mutation profiles of key TP53 pathway components in colorectal cancer, stratified by age of onset (early vs. late) and ancestry (Hispanic/Latino vs. Non-Hispanic White). Panels display mutation types, tumor mutational burden (TMB), and FOLFOX treatment status across: (A) early-onset Hispanic/Latino (H/L) patients, (B) late-onset H/L patients, (C) early-onset Non-Hispanic White (NHW) patients, and (D) late-onset NHW patients.

(A) (B)

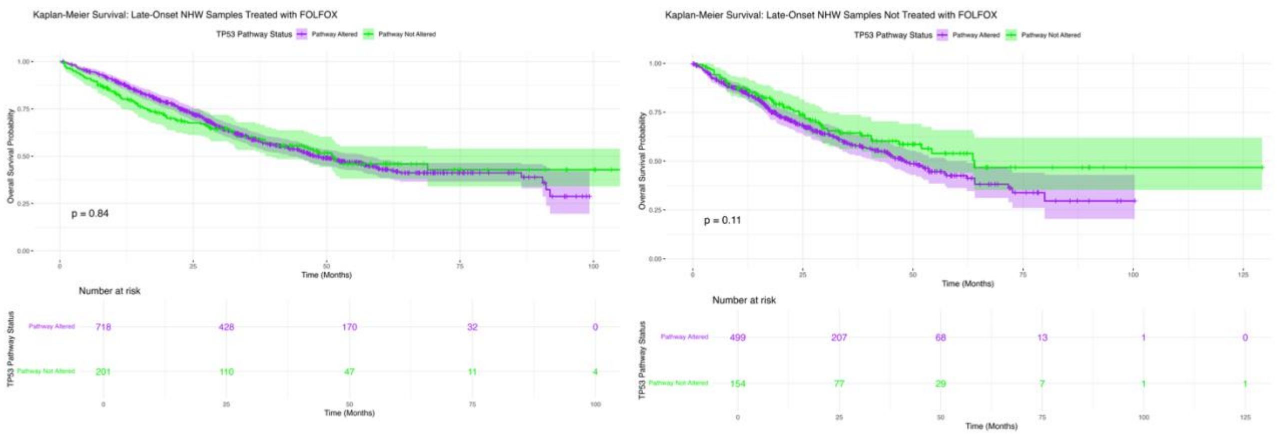

Figure S6. Kaplan–Meier analysis of overall survival by TP53 pathway alteration status in late-onset Non-Hispanic White (NHW) colorectal cancer (CRC) patients. Survival outcomes are stratified according to FOLFOX treatment status, with panels illustrating (A) late-onset NHW patients who received FOLFOX therapy and (B) late-onset NHW patients who did not. Each curve contrasts cases with TP53 pathway alterations against those without, highlighting potential treatment-related survival differences within this subgroup.

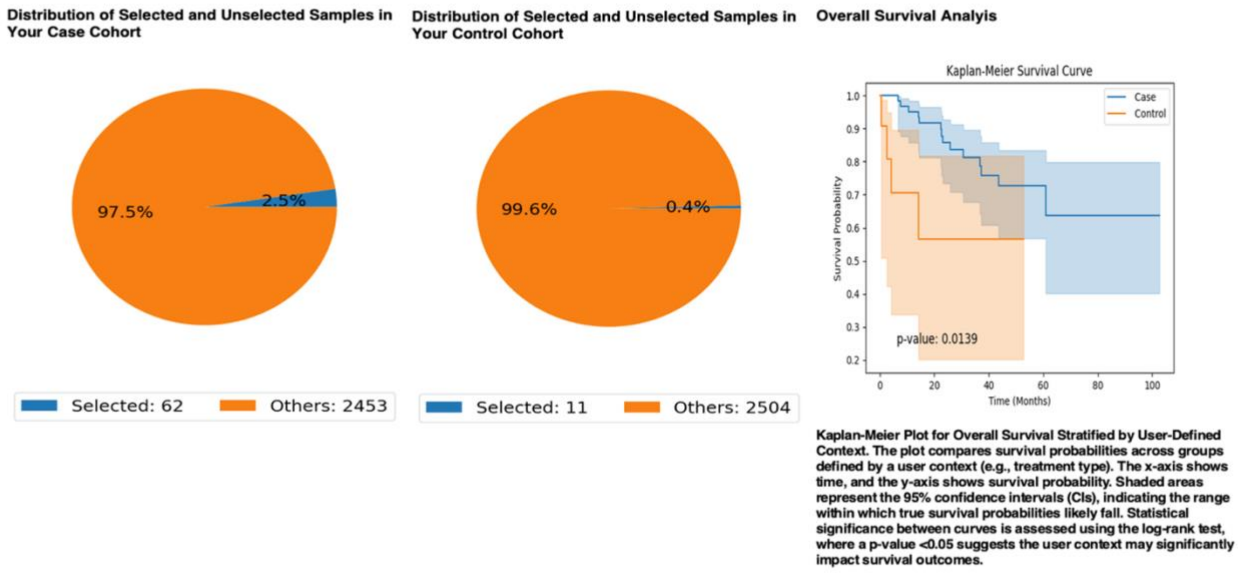

Figure S7. AI-guided selection and survival analysis of early-onset Hispanic/Latino (H/L) colorectal cancer (CRC) patients treated with FOLFOX, stratified by TP53 pathway alteration status. The AI-HOPE and AI-HOPE-TP53 platforms were utilized to define case and control cohorts based on integrative clinical, genomic, and treatment features. (Left) Pie charts illustrate the distribution of selected versus unselected samples for the case cohort—early-onset H/L CRC patients treated with FOLFOX and harboring TP53 pathway alterations (n = 62)—and the control cohort—early-onset H/L CRC patients treated with FOLFOX but without TP53 pathway alterations (n = 11). (Right) Kaplan–Meier overall survival (OS) analysis demonstrates that TP53 pathway–altered cases exhibited significantly reduced survival compared to non-altered controls (log-rank p = 0.0139). Shaded areas represent 95% confidence intervals. The observed separation of survival curves indicates a potential positive prognostic effect of TP53 pathway alterations among early-onset H/L patients receiving FOLFOX chemotherapy, supporting the results of this study.
